# The Andaman day gecko paradox: an ancient endemic without pronounced phylogeographic structure

**DOI:** 10.1038/s41598-020-68402-7

**Published:** 2020-07-16

**Authors:** Ashwini V. Mohan, Pablo Orozco-terWengel, Kartik Shanker, Miguel Vences

**Affiliations:** 1 Department of Evolutionary Biology, Zoological Institute, Braunschweig University of Technology, 38106 Braunschweig, Germany; 2 Centre for Ecological Sciences, Indian Institute of Science, Bangalore, 560012 India; 3 School of Biosciences, Cardiff University, Cardiff, Wales CF10 3AX UK

**Keywords:** Ecology, Evolution, Zoology

## Abstract

The Andaman day gecko (*Phelsuma andamanensis*) is endemic to the Andaman Archipelago, located ~ 6000 km away from Madagascar where the genus *Phelsuma* likely evolved. We complemented existing phylogenetic data with additional markers to show that this species consistently branches off early in the evolution of the genus *Phelsuma,* and this early origin led us to hypothesize that island populations within the Andaman Archipelago could have further diversified. We sampled the Andaman day gecko from all major islands in the Andamans, developed new microsatellite markers and amplified mitochondrial markers to study population diversification. We detected high allelic diversity in microsatellites, but surprisingly poor geographical structuring. This study demonstrates that the Andaman day gecko has a panmictic population (K = 1), but with weak signals for two clusters that we name ‘North’ (North Andaman, Middle Andaman, Interview, Baratang, Neil, and Long Islands) and ‘South’ (Havelock, South Andaman, Little Andaman Islands). The mitochondrial COI gene uncovered wide haplotype sharing across islands with the presence of several private haplotypes (except for the Little Andaman Island, which only had an exclusive private haplotype) signalling ongoing admixture. This species differs from two other Andaman endemic geckos for which we provide comparative mitochondrial data, where haplotypes show a distinct phylogeographic structure. Testing population history scenarios for the Andaman day gecko using Approximate Bayesian Computation (ABC) supports two possible scenarios but fails to tease apart whether admixture or divergence produced the two weak clusters. Both scenarios agree that admixture and/or divergence prior to the onset of the last glacial maximum shaped the genetic diversity and structure detected in this study. ABC supports population expansion, possibly explained by anthropogenic food subsidies via plantations of cash crops, potentially coupled with human mediated dispersal resulting in the observed panmictic population. The Andaman day gecko may thus be a rare example of an island endemic reptile benefiting from habitat modification and increased movement in its native range.

## Introduction

Populations evolve as a result of changing demographic characteristics influenced by habitat components and structure, seasonal availability of resources, individual interactions, and other species-specific variables. While vicariance biogeography posits stable species communities on islands over long periods, island biogeography theory predicts high species turnover as a result of colonization and extinctions, but most empirical studies fall in the intermediate zone with varying levels of geneflow and dispersal across neighbouring islands^1^. The resultant complex demographic histories leave signatures in the genetic makeup of organisms^2^, where the genome functions as an evolutionary memory of populations. Phylogeographic studies, especially on different co-distributed species, can provide insights into historical and contemporary dispersal patterns and gene flow.

Continental islands are islands that share their geological history with a nearby continent and are of several types based on their distance from the mainland and other associated features^3^. Species communities on continental islands are often similar to those on the adjacent mainland, yet differ greatly in genetic diversity and distribution, depending on the time of their separation, geographical proximity to mainland and island size, e.g. Madagascar^4^ versus the Andaman and Nicobar Islands^5^. Madagascar is an isolated large continental island that has been separated by larger sea distances and longer geological times^3^; the Andaman and Nicobar Islands are continental outer arc islands^6^ that have repeatedly been connected to mainland South East Asia during periods of low sea level.

The Andaman and Nicobar Islands are rifted arc-raft continental islands^7^ and part of the Indo-Burma and Sundaland biodiversity hotspots, respectively. These islands are part of a mountain chain extending from Cape Negrais in Burma to Aceh, the northern tip of Sumatra^5,8,9^. The Andaman Islands are separated from each other by shallow sea of no more than 200 m depth^10^, and hence have been repeatedly connected during drops of sea level, most recently in the Late Pleistocene^5^. This archipelago encompasses ~ 300 islands divided into two main groups: the Greater Andamans to the north and Little Andaman to the south, separated by a shallow sea strait named Duncan passage. Most islands in the Andamans share their species assemblages suggesting that the terrestrial species’ populations on different islands have regularly come in contact (see references [5] and [11] for species distributions on different Andaman Islands). Although there have been repeated fluctuations in sea levels, some of which could have connected the Andaman Islands to mainland South-East Asia, these islands still host several endemic lineages, especially in their amphibian and reptile fauna^5,9,11^. The presence of a high proportion of endemic taxa in these islands indicates that terrestrial lineages were separated long enough from their mainland counterparts to have undergone speciation. There are just a handful of studies attempting to understand the effects of relatively recent island separation on populations of endemic terrestrial fauna in the Andaman Islands. Phylogeographic studies on four bat species^12^ and the Andaman keelback snake (*Fowlea tytleri* (Blyth, 1863))^13^ showed substantial levels of genetic diversity, structure and varying degrees of inter-island population differentiation in mitochondrial DNA.

The Andaman day gecko (*Phelsuma andamanensis* Blyth, 1861) is an exception to the biogeographical origins of endemic lineages in the Andaman Islands. This species, endemic to the Andaman Islands, belongs to a genus that likely evolved in Madagascar, ~ 6,000 km westwards across the Indian Ocean^14^. Among the Western Indian Ocean islands colonized by *Phelsuma*, the Seychelles are the closest to the Andamans in distance, yet the two lineages from the two archipelagos are not closely related in the *Phelsuma* phylogeny^14^, but phylogenetic relationships between the deeper *Phelsuma* clades remain largely unresolved^14^. The partial DNA sequences from a few genes that have so far been used have rendered insufficient information to reliably reconstruct the evolutionary history of the genus, possibly due to rapid radiation not providing enough time for accumulation of genetic mutations and/or extinctions of key links between the main clades. However, studies have consistently found that the Andaman day gecko represents one of the oldest lineages in the genus *Phelsuma*^14–17^, i.e., it splits from one of the most basal nodes in the phylogeny without any closely related sister species. Some of these studies had extensive taxon sampling, representing almost all known species in the genus Phelsuma^17^ and therefore are unlikely to result from unresolved relationships as a result of missing taxa. In this study, we aim to resolve deep clade relationships within the genus *Phelsuma* by amplifying additional nuclear and mitochondrial markers. This is important as a species’ biogeographic origin has a key role in its phylogeographic and population genetic processes.

*Phelsuma* day geckos have diversified in all the archipelagos they have reached^17–20^, but it is not known whether the Andaman lineage diversified after colonizing these islands. In contrast, the Mascarene Islands’ *Phelsuma* geckos provide a good example of diversification after colonization with one common ancestral lineage giving rise to nine endemic species, two of which are now extinct^15,19^.

**Figure 1:**
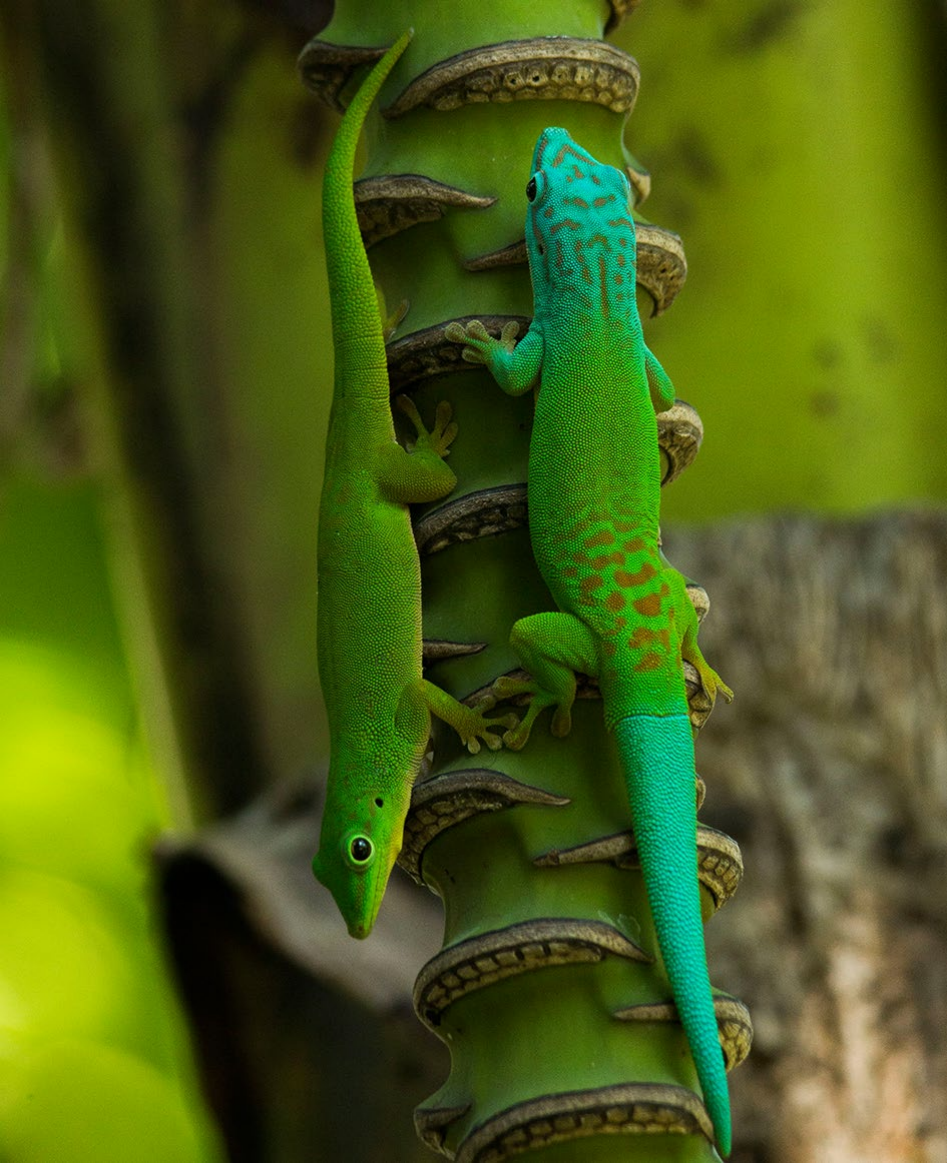
Female (evenly green) and male (turquoise head and tail) individuals of the Andaman day gecko (*Phelsuma andamanensis*) from the North Andaman Island. Image from Sanjay Prasad used with permission.

The Andaman day gecko inhabits tropical forests as well as human made banana (*Musa* sp.), coconut (*Cocos nucifera*) and betel nut (*Areca catechu*) plantations^21^. Such diversity of habitat and colonization of human modified areas is also known from *Phelsuma* species on other islands^18,22^. In fact, coconut plantations host the highest observed population densities of *P. andamanensis* as these areas are rich in food resources such as insects that are attracted to the flower nectar^21^. Densities were observed to be lower in forests dominated by hardwood species, but this might be due to observation bias^21^. This species is diurnal and sexually dimorphic; males are larger in size with a bluish-turquoise tail and females smaller and are evenly green in colour^21^ (Fig. 1).

While several species of *Phelsuma* have been studied in terms of diversification at the level of species and populations^23–25^, the Andaman day gecko remains poorly studied. Considering the adaptability and higher densities of the Andaman day gecko in plantations, we hypothesize that these plantations could have facilitated population expansion in larger, human-inhabited islands of the Andamans. We aim to assess genetic diversity within the Andaman day gecko lineage and test various scenarios of population division, admixture and to calculate population demographics. Considering the genetic diversity observed in similar spatial scales on the Seychelles, we hypothesize that the Andaman day gecko has genetically structured populations across the Andaman Islands. In order to address this question, we explore both mitochondrial DNA sequences and nuclear microsatellite markers that were specifically developed for this study. Besides classical descriptive phylogeography analyses^26^, we also apply more recent methods of statistical phylogeography which allow testing of explicit models of population history using population demographic parameters, with Bayesian Markov Chain Monte Carlo (MCMC) approaches^27^. Specifically, we apply Approximate Bayesian Computation (ABC) analysis jointly on microsatellite markers and mitochondrial DNA sequences to elucidate the demographic history of the Andaman day gecko.

Comparative phylogeography has been key to understanding how lineages of different taxa respond to common geographical and geological events. Ancestrally co-distributed biota, which have undergone similar vicariant events are expected to show similar spatial structuring of populations^28^. Other Gekkonidae in the Andaman Islands include endemic species from South-East Asian genera like *Cyrtodactylus* and *Gekko*; and introduced gecko species with largely oceanic distributions like the common house gecko (*Hemidactylus frenatus* Dum $\acute{e}$ ril and Bibron, 1836) and the four-clawed gecko (*Gehyra mutilata* (Wiegmann, 1834))^5^. The endemic gecko species almost always occur in similar natural and human-modified habitats as the Andaman day gecko, but differ in microhabitat use and time of activity. The Andaman day gecko is an arboreal, diurnal species; the Andaman Giant gecko (*Gekko verreauxi* Tytler, 1865) is arboreal but nocturnal; and the Andaman bent-toed gecko (*Cyrtodactylus rubidus* (Blyth, 1861)) is a ground dwelling species, often found in lower parts of thick trunks of native trees and vespertine in activity. Extending our main focus on the Andaman day gecko, we also compare the mitochondrial phylogeographies of these three gecko species, to understand whether the patterns observed in *P. andamanensis* are singular for this species, or common to Andaman endemic squamates similarly influenced by geographical, geological and anthropogenic factors.

## Results

### Phylogenetic position of the Andaman day gecko

**Figure 2:**
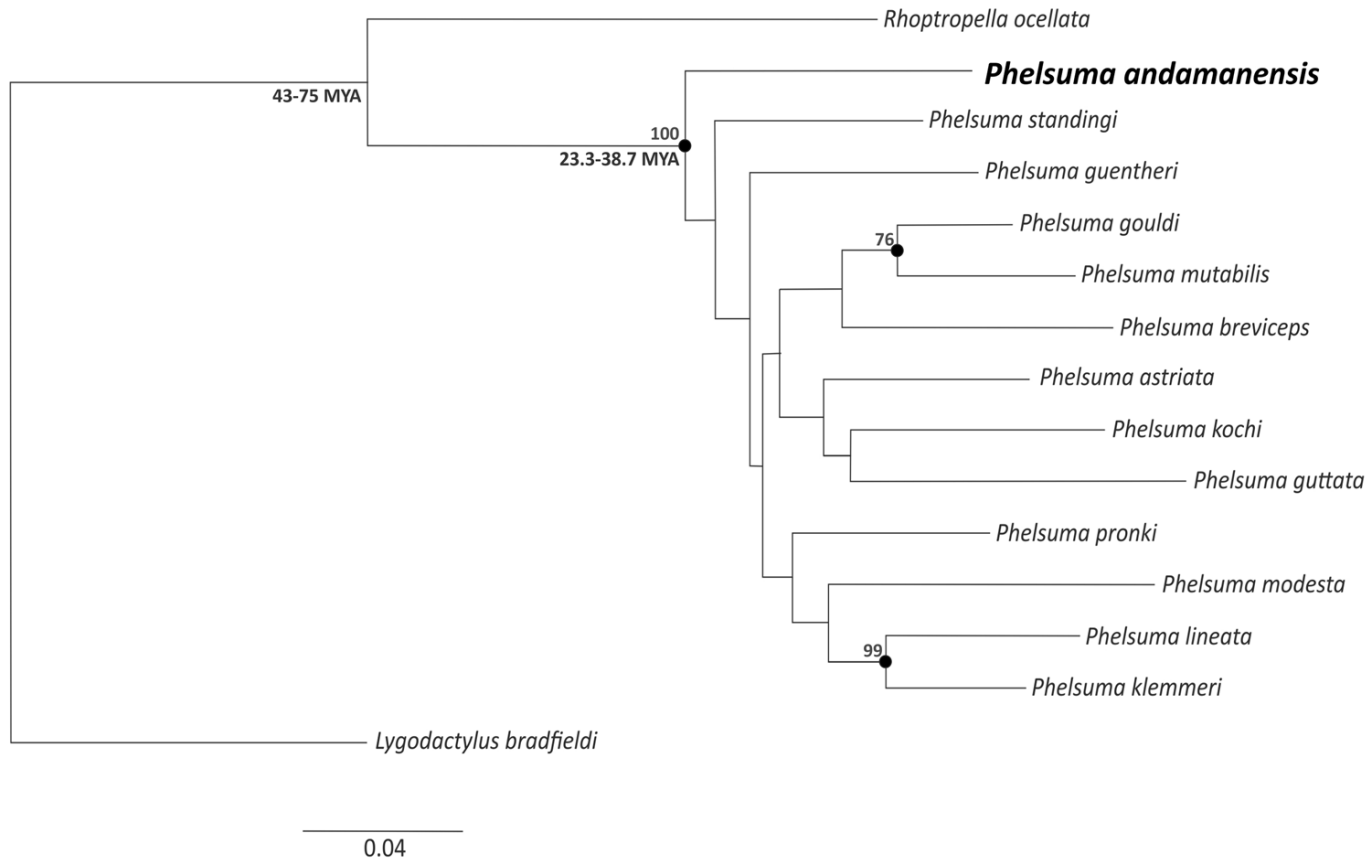
Maximum likelihood analysis of a concatenated alignment of 9,278 bp from 4 mitochondrial and 9 nuclear genes run 10 times, with 10,000 bootstrap replicates each. Black dotted nodes represent Maximum Likelihood (ML) supported nodes with a support above 70% (other nodes received less support). Numbers at the first two nodes are pairwise divergence time estimates (confidence intervals) obtained from Timetree.org, shown as general indication of the likely time frame of *Phelsuma* diversification. Figure labelled using CorelDraw Home & Student, X7 (https://www.coreldraw.com).

A molecular phylogeny reconstructed using four mitochondrial and nine nuclear genes from 15 species including the Namaqua day gecko [*Rhoptropella ocellata* (Boulenger, 1885)] and Bradfield's Dwarf Gecko (*Lygodactylus bradfieldi* Hewitt, 1932) as outgroups confirms that the Andaman day gecko has an isolated phylogenetic position (Fig. 2). It splits from one of the most basal nodes in the phylogeny as shown in previously published phylogenies of the genus *Phelsuma*^14–17^. However, we find that this phylogeny is sensitive to change in calculation parameters which reflects the lack of support in establishing deeper relationships in the genus *Phelsuma*. While a thorough time calibration of the *Phelsuma* tree is beyond the scope of the present paper, previous studies (e.g.,^29^) placed the *Phelsuma* crown age at around 30 million years (MYA), and the TimeTree database (timetree.org; accessed 19 May 2020) estimated the divergence between the Andaman day gecko and the Madagascar day gecko (*Phelsuma madagascariensis* Gray, 1831) at ~ 27 MYA^30^, suggesting that the Andaman day gecko is an ancient lineage.

### Population genetics of the Andaman day gecko

From a newly developed microsatellite library for the Andaman day gecko, we obtained data from 140 samples and 13 markers to analyse population genetic diversity and structure. Fisher’s pairwise comparison of microsatellite markers to test linkage disequilibrium or genotyping association within island populations showed no significant linkage between any pairs of markers (FDR_Benjamin-Yekutieli_ threshold *p* = − 0.010121; see Supplementary S2, Table 1). Genetic diversity was high, with an average allele number of 23.7 and a mean expected heterozygosity of 0.92 (Table 1) and 7.4% of data from all 13 loci was missing/not usable.

**Table 1:** New Andaman day geckos (*Phelsuma andamanensis*) microsatellite primers

Marker name	Forward primer	Reverse primer	Motif	Expected PCR product size	Observed range of PCR product size
Pand_micro2	TTTGAGCAGGCAAGACAGAC	AAGGTGCTAGACTAGATGGACC	(AAAG)_13_	193	175–288
Pand_micro6	GCTCTATGAGAACCGCTATCAG	TGGTGGAGGCTAATGAGGTATG	(AATC)_11_	197	217–273
Pand_micro8	GAGGGAGACAACCAAGGAATAC	GCTTTCTCTCTGCCTCATATGG	(AAAG)_11_	242	244–350
Pand_micro10	GCATGAAGATGAGGTTAGAGGG	CAGTTGATCCAGAGCTGTGTTC	(AAAG)_11_	225	247–346
Pand_micro12	TCTTAGGCCCTATAACTCCTCC	CAGCCCGTTGTCTTAGAGTATC	(AAAG)_14_	250	279–383
Pand_micro13	CTCTTTGGACTCTGGACTCATG	CCCATCCAAATACTAACCAGGG	(ACAT)_10_	192	211–263
Pand_micro15	CAAGGTCTCCATGCATGTGTAC	TATTCTAGGGAGGACAGGAACC	(ATCC)_12_	222	226–338
Pand_micro16	CCTTCTCCTTTGGTGTACGATG	CAACCAATGACTGCTAGGGAAG	(AAAC)_11_	153	157–204
Pand_micro17	GTGAAAGTCCATCTACTGTGGC	GTAGTGGTTAAGAGTGGCAGTC	(ACAG)_15_	182	170–383
Pand_micro19	CGGATGTCTACAGTCTGAAGTG	GGGCTCAGTAGTATAAGACCTG	(AAAG)_14_	241	230–293
Pand_micro23	GTCAGACAATGTGAGGATCTCC	CTCCTCCAAGTCATCTCCAAAC	(AATC)_13_	172	177–272
Pand_micro4	TACCCACCATAGCTCCTTAG	AGGAGTCCTTTGGTACCTTG	(AAAG)_13_	155	165–248
Pand_micro9	CTGCCTTAATCTAGAAGCCCTC	TAAGAGGGTAGGGTGGGTATTG	(ATCC)_15_	215	207–274

Forward primer and reserve primer columns provide the primer sequence and Motif shows the 4 bp repeat motif of each locus. PCR product size is the expected value from the primer design procedure.

The number of microsatellite alleles detected from each sampled island was correlated with sample size (see Supplementary S2, Fig. 1 online), indicating that increased sampling efforts could detect a greater number of alleles. Missing data was on average 9% per locus varying in the range between 0 and 60% with the highest percentage shown in the marker Pand9. Three of the 13 tested markers deviated significantly from Hardy Weinberg Equilibrium (FDR_Benjamin-Yekutieli_ threshold *p* = 0.015723; Supplementary S2; Table 2 online). Apart from the global Hardy Weinberg test of markers, each marker was also tested for Hardy–Weinberg Equilibrium in every island population (see Supplementary S2, Table 3 online). This test showed that only the Middle and North Andaman populations have one and two microsatellite markers significantly deviating from HWE (FDR_Benjamin-Yekutieli_ threshold *p* = 0.009357; see Supplementary S3, Table 3), respectively. Null alleles were detected in one marker (see Supplementary S2, Table 5 online), Pand9, which was also one of the markers with high levels of missing data and therefore inferences about presence of null alleles need to be carefully interpreted.

**Table 2:** Genetic variation summary statistics

Locus	Whole dataset			North Andaman (n = 47)		Middle Andaman (n = 50)		Havelock (n = 9)		Baratang (n = 11)		South Andaman (n = 12)		Little Andaman (n = 6)	
	NA	He	Ho	He	F_IS_	He	F_IS_	He	F_IS_	He	F_IS_	He	F_IS_	He	F_IS_
Pand2	27.00	0.92	0.75	0.93	0.12	0.91	0.22	0.78	0.29	0.93	**0.00**	0.92	0.36	0.95	**− 0.10**
Pand6	16.00	0.88	0.79	0.86	0.08	0.90	0.07	0.85	0.20	0.86	0.03	0.85	0.10	0.89	0.43
Pand8	25.00	0.94	0.84	0.94	0.03	0.93	0.14	0.95	0.04	0.93	0.10	0.92	0.17	0.92	0.06
Pand10	26.00	0.95	0.88	0.94	**− 0.03**	0.95	0.13	0.92	**0.00**	0.95	**− 0.08**	0.94	0.28	0.95	**− 0.10**
Pand12	29.00	0.95	0.82	0.93	0.20	0.94	0.09	0.91	**− 0.01**	0.91	**− 0.02**	0.93	**0.00**	0.88	0.42
Pand13	17.00	0.91	0.81	0.89	0.13	0.92	0.13	0.90	**− 0.02**	0.91	**− 0.02**	0.90	**− 0.04**	0.73	**− 0.21**
Pand15	26.00	0.94	0.80	0.94	0.15	0.93	0.16	0.93	**0.01**	0.91	**− 0.02**	0.91	0.16	0.94	0.27
Pand16	14.00	0.84	0.74	0.83	0.07	0.86	0.13	0.80	0.44	0.82	**− 0.02**	0.78	**− 0.09**	0.56	0.39
Pand17	49.00	0.96	0.88	0.97	0.06	0.96	0.13	0.97	0.18	0.94	**− 0.09**	0.97	0.12	0.98	**− 0.06**
Pand19	17.00	0.93	0.87	0.93	0.09	0.93	0.02	0.91	0.25	0.94	**0.01**	0.95	0.02	0.88	**0.01**
Pand23	20.00	0.91	0.83	0.92	0.16	0.90	0.05	0.85	**− 0.08**	0.93	**0.00**	0.89	0.24	0.92	**− 0.14**
Pand4	23.00	0.93	0.79	0.92	0.11	0.93	0.10	0.84	0.33	0.93	0.20	0.90	0.16	0.88	**0.01**
Pand9	19.00	0.84	0.53	0.83	0.53	0.90	0.18	0.68	0.33	0.86	0.25	0.31	0.45	0.73	0.53
Overall	23.69	0.92	0.79	0.91	0.13	0.92	0.12	0.87	0.15	0.91	0.03	0.86	0.15	0.86	0.12

Summary statistics of the genetic variation across markers for island populations with more than two samples. NA = Number of Alleles, He = Expected heterozygosity, Ho = Observed heterozygosity, F_IS_ = Inbreeding Coefficient. Values in bold are statistically significant, inferred using FDR correct p value (*p* = 0.009356887)

**Table 3:** Population pairwise F_ST_ calculated using microsatellite and mitochondrial (COI + 16S) data

NorA		0.02	− 0.02	− 0.01	0.01	0.03	0.24	0.01	0.03
MA	0.00		0.06	− 0.02	0.03	0.03	0.09	0.02	0.08
Int	0.00	− 0.01		0.11	0.06	0.16	1.00	0.08	0.08
Long	0.00	− 0.01	**0.00**		− 0.06	− 0.01	0.11	− 0.04	0.08
BA	0.00	0.01	0.03	0.01		0.04	0.26	0.01	0.08
HA	0.01	**0.02**	0.02	0.03	0.01		0.16	− 0.01	0.11
Neil	− 0.03	− 0.02	**0.02**	0.01	− 0.03	0.01		0.20	0.49
SA	**0.02**	**0.03**	0.04	0.04	0.02	0.02	− 0.02		0.07
LA	**0.02**	**0.03**	0.02	0.03	0.02	0.01	0.01	**0.02**	

F_ST_ values between populations estimated from the microsatellite data are shown in the lower left half of the table, while G_ST_ values estimated from the mitochondrial DNA are shown on the upper right side. Samples are grouped based on the island of origin and abbreviated as NorA: North Andaman, MA: Middle Andaman, Int: Interview, Long: Long Island, HA: Havelock, BA: Baratang, Neil: Neil Island SA, South Andaman and LA: Little Andaman. Values highlighted in the table correspond to significant divergences after FDR correction (FDR corrected *p* = 0.01198).

Overall, pairwise divergence between populations based on the microsatellite data was low (F_ST_ = 0.009923, *p* = 0.0001) but statistically significant, indicating island populations show a certain degree of differentiation. Population pairwise F_ST_ suggested the levels of differentiation are low, with 0.04 being the highest, between South Andaman and Long Island, Interview Island (Table 3). The low Fixation index (F_ST_) value could be due to high allele diversity in the microsatellite markers as this metric was designed for bi-allelic data^31^.

**Figure 3:**
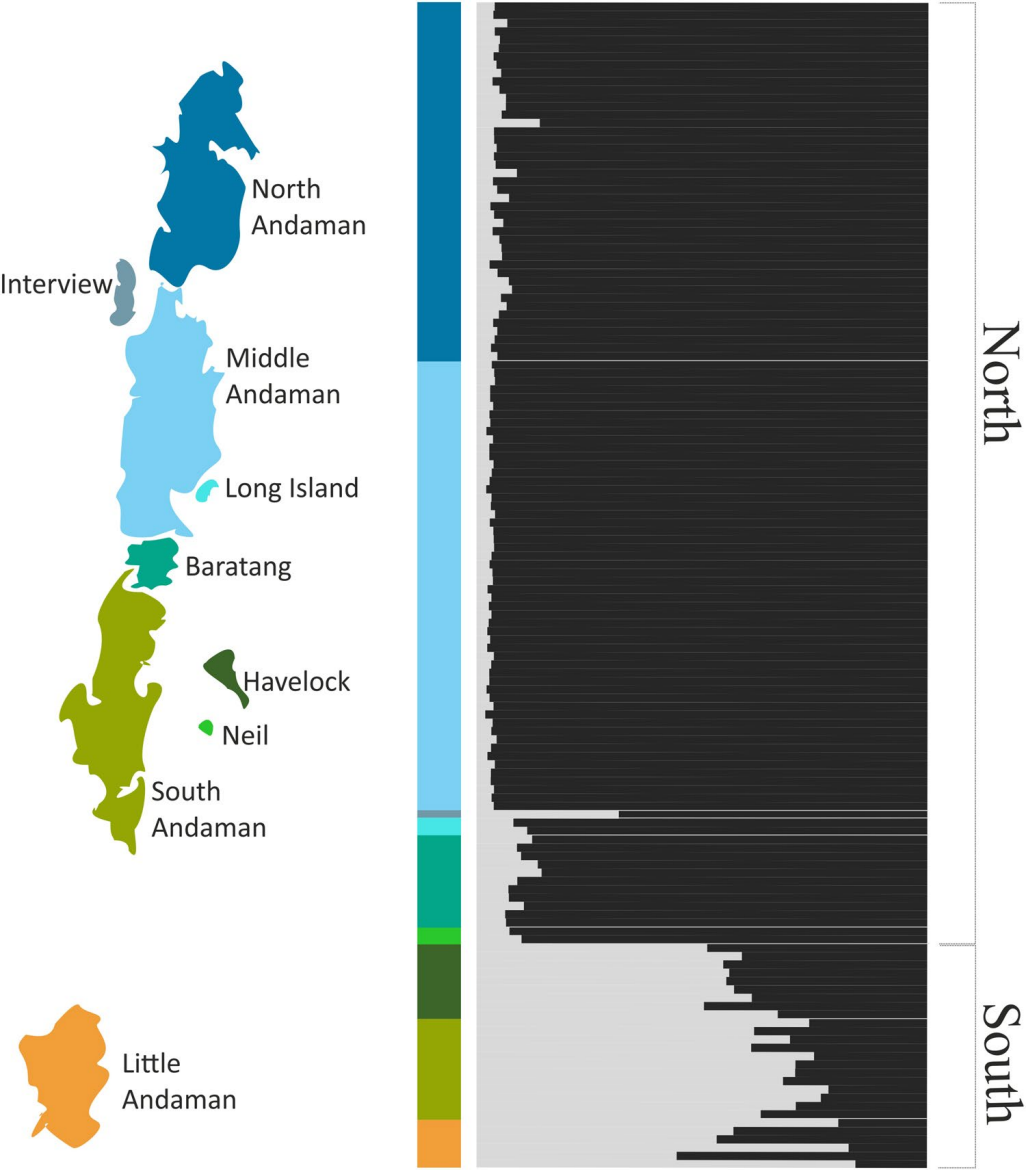
Population clusters identified using microsatellite alleles of the Andaman day gecko (*Phelsuma andamanensis*) (K = 2). Individuals are colour coded based on the island of their origin. North and South clusters are identified on the right. Composite figure produced in CorelDraw Home & Student, X7 (https://www.coreldraw.com), map created for graphical representation only and is not to be scaled.

**Figure 4:**
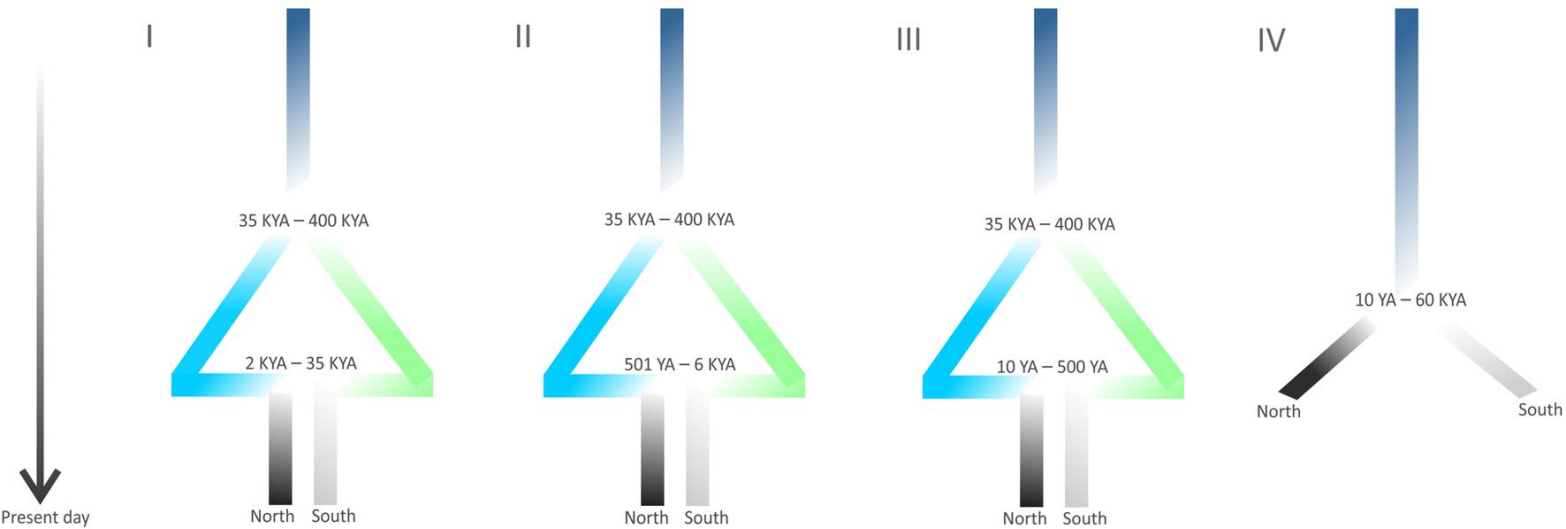
Schematic of the four demographic models tested to explain the North and South clusters identified with the nuclear markers. Time is measured from the past (top) towards the present (bottom). Scenario I, II and III consist of an ancestral population (dark blue) that splits into two derived populations sometime between 35,000 years ago and 400,000 years ago giving rise to the precursors of the northern and southern genetic clusters. The difference between these three scenarios is the time at which an admixture event, with equal contributions of both populations, occurred between the two ancestral precursor clusters and which resulted in the current North and South clusters. Scenario IV consisted of an ancestral population splitting into two forming the North and the South cluster sometime between 10 YA and 60 KYA. KYA: time of divergence in thousands (kilo) years ago. YA: years ago. Figure produced in CorelDraw Home & Student, X7 (https://www.coreldraw.com).

Cluster analysis with the program Structure^32^ using (1) all 13 microsatellite markers, (2) removing the marker Pand9 (as it showed presence of null alleles and has high levels of missing data; see Supplementary S2, Fig. 3 online), consistently resulted in one cluster (K = 1) having the highest likelihood of explaining the data (see Supplementary S2, Fig. 4 online). This result was also supported by re-running another clustering algorithm using both admixture and no-admixture models implemented in rmaverick^33^ (see Supplementary S2, Fig. 4 online). Therefore, both Structure and rmaverick identified the best possible K as 1. However, at K = 2 using a location prior model in Structure^34^, individuals sorted into two geographically meaningful clusters with a posterior probability of > 0.50 showing that K = 2 is the second-best scenario to explain the contemporary population structure. In other words, K = 2 shows that at the highest hierarchical level, the Andaman day gecko has a weak structure of two populations occurring in the northern and the southern islands, hereon referred to as ‘North’ and ‘South’ clusters. The North cluster contains all islands except Havelock, South Andaman and Little Andaman (Fig. 3).

**Figure 5:**
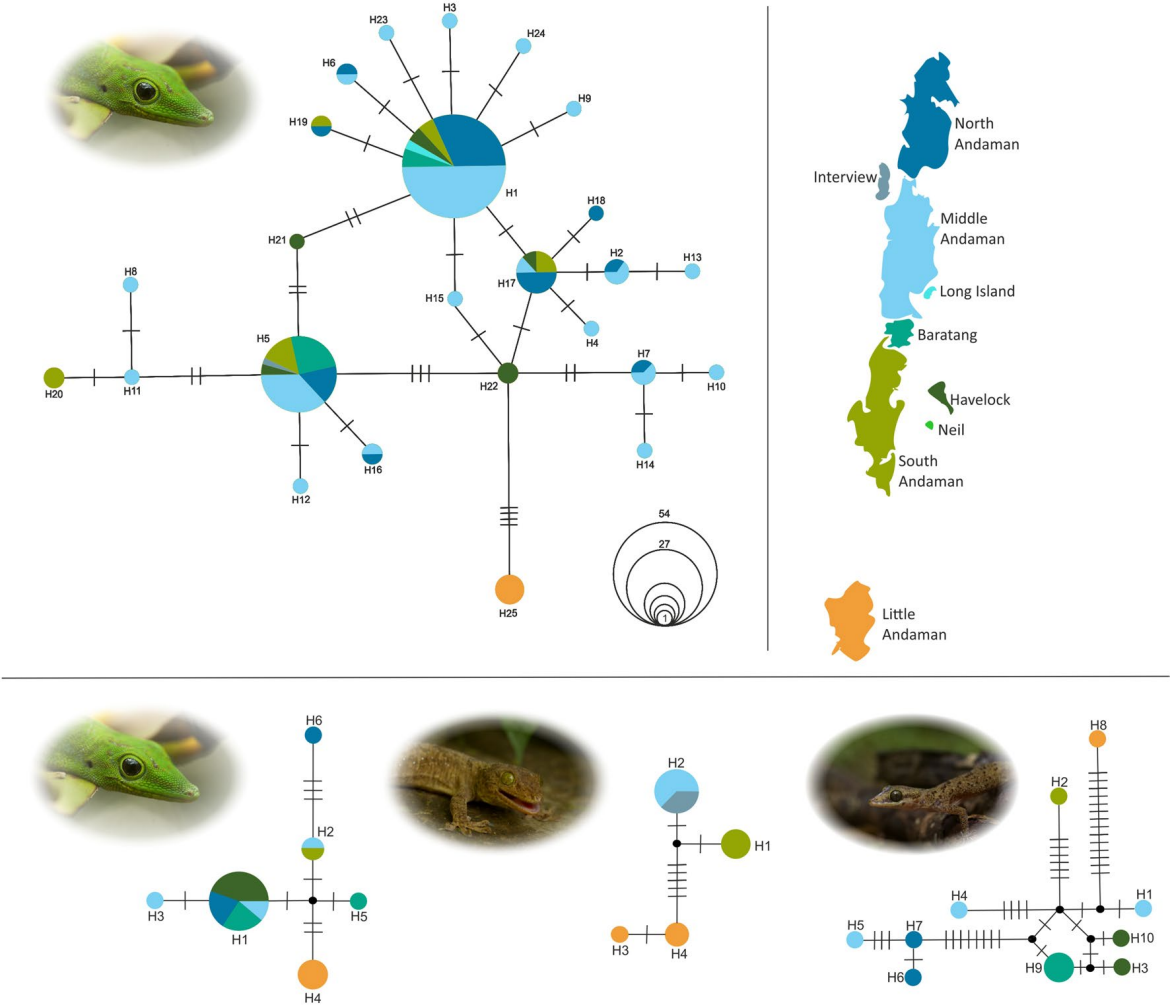
Top: Haplotype network from DNA sequences of 314 bp of the mitochondrial COI gene for 123 individuals of the Andaman day gecko (*Phelsuma andamanensis)* sampled across the Andaman Islands. Haplotypes are colour coded based on the island on which an individual was sampled; the size of the circles in the haplotype network corresponds to haplotype frequencies whose scale is provided in uncoloured circles; each cross bar in the network represents a mutation and black points correspond to median vectors generated during the construction of the Median-Joining haplotype network. Bottom: Haplotype networks from DNA sequences of the mitochondrial 16SrRNA from three species of gecko endemic to the Andaman Islands. left: the Andaman day gecko (*Phelsuma andamanensis*); centre: the Andaman bent toed gecko (*Cyrtodactylus rubidus*); right: the Andaman giant gecko (*Gekko verreauxi*). Composite figure produced in CorelDraw Home & Student, X7 (https://www.coreldraw.com), map created for graphical representation only and is not to be scaled.

We subjected the dataset to clustering at higher values of K, i.e. K > 2 to determine how individuals segregate with progressively larger values of the number of clusters (see Supplementary S2, Fig. 5), but they showed no clustering. Microsatellite markers evolve at a faster rate than mitochondrial markers and hence they may not be able to detect more ancient historical population structure, even if traces of such structure may still be recognizable in the distribution of mitochondrial haplotypes.

### Testing best fit population demographic models through Approximate Bayesian Computations (ABC)

The molecular analyses of the Andaman day gecko described above suggested (1) absence of strong phylogeographic structure both from mitochondrial DNA and microsatellites, (2) limited divergence among mitochondrial haplotypes, and (3) a signal for two weakly defined and possibly geographically admixed genetic clusters (North and South). Informed by these analyses, we defined four evolutionary and demographic scenarios (Fig. 4) and tested these using ABC models by incorporating both microsatellite and mitochondrial sequence data. Microsatellite data included 13 microsatellite markers, sequenced from 140 individuals of the Andaman day gecko, whereas the mitochondrial data comprised COI sequences from 123 individuals. These four alternative demographic scenarios were compared to determine the most likely ancestral demographic history explaining the weak signal of a partition into two clusters.

Among the four scenarios tested, scenarios II and III received support of less than 2%, indicating that these two weak clusters were a result of older events. Scenarios I and IV had a similar statistical support [~ 47.7% (95% CI 4.8–50.7%) and 51% (95% CI 48.2–53.8%), respectively] using the logistic regression approach implemented in DIY-ABC. However, the Direct Approach (a count of the occurrences of each model across the most similar simulations to the observed data) indicates that scenario I is slightly more likely. On the other hand, the probabilities, and 95% CIs of scenarios I and IV are largely overlapping [52% (95% CI 9–96.6%) and 35.6% (95% CI 0–77.6%), respectively]. While this implies that scenario II and III are not likely to describe the genetic variation observed in this study, there is no clear evidence to distinguish between the scenarios I and IV (Fig. 4, Table 3). The main difference between scenarios I and IV was the presence of admixture in scenario I that does not feature in scenario IV. Overall, the parameter estimations for both scenarios are quite similar (Table 3) with the North cluster having a larger effective population size than the South cluster. In both the scenarios, the North and South clusters have a larger effective population size than that of their ancestral populations indicating a population expansion post their divergence. In fact, the time of admixture inferred from scenario I (~ 27,400 years ago—95%CI 884–34,400 years ago) is largely overlapping with the estimated time of divergence of the simpler model in scenario IV (~ 23,600 years ago (95%CI 711–55,900 years ago). Consequently, it is likely that the statistical power to infer evolutionary history further in the past, i.e., beyond the most recent divergence/admixture event affecting the North and South genetic clusters is small with this dataset.

### Comparative phylogeography of Andaman endemic Gekkonidae

The 314 bp mitochondrial DNA sequences of the cytochrome oxidase subunit I gene (COI) from 123 Andaman day gecko (*P. andamanensis*) individuals detected 25 haplotypes with an overall nucleotide diversity (Pi) of 0.0071 and haplotype diversity (H_d_) of 0.756. Maximum uncorrected *p* distance among COI sequences was 1.6% between individuals sampled on Baratang and Little Andaman. We found 28 polymorphic sites and a total of 29 different mutations. Among the 25 recognized COI haplotypes, 19 were detected in individuals from the Middle Andaman and among these, 12 were private alleles (Fig. 5). All individuals from the Little Andaman showed a single haplotype, private to that island (Fig. 5). The North Andaman and South Andaman island populations had one private allele each, in addition to common haplotypes that were detected in other island populations. From individuals on Havelock, we detected two private alleles and five haplotypes in total. Among the individuals sampled in the islands of Baratang, Long and Interview, we only detected common haplotypes on Middle Andaman and other islands.

To allow direct comparability with other geckos for which only sequences of the mitochondrial 16S rRNA gene were available, we sequenced a 463 bp segment of this gene for 17 individuals of the Andaman day gecko. We detected six different 16S haplotypes (Fig. 5), four of which were private alleles from isolated human settlements in the northern part of the North Andaman, Little Andaman, Middle Andaman and Baratang, respectively. H1 was the most frequent haplotype detected from four islands and the maximum uncorrected *p* distance was 0.9% (see Supplementary S2, Table 4 online). Although this may seem a low number of haplotypes when compared to COI, the probability of observing 6 haplotypes in a sample of 17 COI sequences is ~ 40%, indicating that both markers harbour similar levels of diversity (see Supplementary S2, Fig. 2 online).

**Table 4:** Posterior probabilities for each of the demographic parameters of the scenarios one and four

Parameter	Scenario 1			Scenario 4		
	Mode	q025	q975	Mode	q025	q975
N1	2.42e6	1.14e6	7.81e6	3.74e6	1.60e+006	7.85e+006
N2	3.34e5	1.33e5	9.45e5	4.19e5	1.52e+005	9.51e+005
N3	1.16e4	3.91e3	3.37e5			
N4	2.16e4	6.75e3	3.42e5			
t-admix	2.76e4	8.84e3	3.44e4			
t-div	1.53e5	4.84e4	3.84e5	2.36e4	7.11e+003	5.59e+004
NA	1.63e4	9.28e3	8.03e5	2.14e4	2.14e+004	4.65e+005
useq_1	1.78e−8	1.2e−8	1.54e−7	1.11e−008	1.09e−008	3.51e−008
k1seq_1	6.41e0	1.54e0	2.87e1	5.28e+000	1.14e+000	2.79e+001
µmic_1	1.00e−4	1.00e−4	2.4e−4	1.00e−004	1.00e−004	2.17e−004
pmic_1	1.00e−1	1.00e−1	2.72–1	1.00e−001	1.00e−001	2.67e−001
snimic_1	1.00e−8	1.00e−8	2.31–6	1.00e−008	1.02e−008	8.85e−007

95% confidence interval range are shown as lower boundary q025 (2.5%) and upper boundary q0975 (97.5%). N1 is the North population effective population size and N2 is that of the South population. N3 is the precursor population of the North population (before admixture) and N4 is the precursor population of the South population (before admixture). t-admix is the time of admixture and t-div is the time of divergence between the north and south lineages. NA is the ancestral effective population size. useq is the mitochondrial sequence mutation rate, k1seq is the number of haplotypes, µmic is the microsatellite mutation rate, and pmic and snimc are the larger than one step proportion of mutations and the non-standard mutation rate (e.g. indels).

Contrastingly, from 13 individuals of the Andaman bent-toed gecko (*Cyrtodactylus rubidus*) we detected 10 haplotypes in a 480 bp segment of the 16S rRNA gene, none of which were widespread, and all sampled islands showed private alleles (Fig. 5). The maximum pairwise uncorrected *p* distance calculated from this taxon’s 16S sequences was 1.83% (see Supplementary S2, Table 4 online). Not only did the islands all have unique haplotypes, but they were all more genetically distinct from each other than the haplotypes of the Andaman day gecko; for instance, the haplotype detected on Little Andaman Island had 14 bp differences from the closest median vector (Fig. 5) compared to 2 bp difference in the Andaman day gecko (Fig. 5).

From 12 individuals of the Andaman giant gecko (*Gekko verreauxi*) sequenced for 516 bp of the 16S rRNA gene, we found four haplotypes with a maximum uncorrected *p* distance of 1.16% (see Supplementary S2, Table 4 online). Three of these haplotypes were private, two of them being from Little Andaman Island (Fig. 5).

The visualization of genetic structure obtained from mitochondrial markers and microsatellite markers across different island populations resulted in non-concordant results (see Supplementary S2, Fig. 7 online). This is expected considering differences in their inheritance pattern and rate of evolution. While the COI dataset suggested an area of population differentiation between the the Greater Andamans and Little Andaman, the pairwise comparison values in the microsatellite allele sharing matrix suggests geographically non-structured allele sharing (see Supplementary S2, Fig. 7 online) concordant with a panmictic population. In other words, mitochondrial DNA suggests that the population on the Little Andaman Island shows the highest difference from the rest of the island populations in the Andamans. On the other hand, the microsatellite markers suggests that populations are quite admixed with comparable sequence divergences across all the Andaman Islands.

## Discussion

The updated molecular phylogeny of the genus *Phelsuma* presented herein confirms that the Andaman day gecko is one of the oldest lineages in this genus (Fig. 2). Our molecular phylogeny even suggests that it is sister to all other clades of *Phelsuma* spp*.*, However, a full taxon sampling and more genetic data are necessary to resolve these deep relationships with more confidence. We do note that the isolated position of *P. andamanensis* is also supported by an ongoing phylogenomic study (A. Mohan, M. Vences, unpubl. data). This indicates that the ancestors of this species may have originated in Madagascar, as is likely for other major clades of this genus^16,24^. Nevertheless we do not draw biogeographical inferences from our phylogenetic tree, rather use it to illustrate that the Andaman day gecko is an old lineage whose divergence approximates the crown age of *Phelsuma* diversification, which in previous studies was estimated between 43–35 MYA(Fig. 2).

The sheer number and variation of mitochondrial haplotypes detected in the Andaman day gecko refutes the possibility of a non-Andamanese origin and human mediated introduction of this lineage to the Andaman Islands (from an as of yet unknown, or extinct, source population elsewhere). The early humans of the Andaman Islands arrived via Southeast Asia less than 26,000 years ago^35,36^. Despite the uncertainties in the human colonization history of the Andaman Islands (via South Asia or South-East Asia), we see no possibility for human-mediated introduction of this species to the Andaman Islands. Therefore, our study strongly suggests that the genus *Phelsuma* colonized the Andaman Islands naturally, without the aid of humans.

Microsatellite markers developed in this study are a valuable resource to study and monitor the genetic variation in the Andaman day gecko due to the high polymorphism and largely independent segregation of these markers. We interpret the observed high polymorphism as reflecting population demographic processes rather than linkage in the genome. Among microsatellite markers, markers with null alleles are known to be potentially informative in analysing number of clusters and population structure^37^. In our study, the inclusion of the one marker that may include null alleles did not affect the results (see Fig. 3 vs. Supplementary S2, Fig. 3 online). Testing island populations for HWE indicated that only North and Middle Andaman populations had one and two markers that deviated from HWE. Even though results of allele distribution and richness could change with sampling in the Jarawa Tribal Reserve (northern parts of the South Andaman), they will not reduce the recognized levels of diversity and differentiation in this study.

Microsatellite markers suggest that the Andaman day gecko currently has a single panmictic population on the Andaman Islands, as indicated by a high likelihood value for K = 1 in Structure (see Supplementary S2, Figs. 4, 5 online). Model comparison between admixture and no-admixture models in rmaverick did not provide clear support to either of the models indicating that there is neither clear admixture nor complete lack of admixture between island populations. This could also reflect a complex population demographic scenario such as repeated admixture and vicariance in the past. The second-best partition (K = 2) detected two poorly differentiated, yet geographically meaningful clusters in Structure (Fig. 3). The ’South’ cluster consists of individuals sampled on South Andaman, Little Andaman, and Havelock; the ‘North’ cluster consists of individuals sampled on North Andaman, Middle Andaman, Interview, Long, Neil and Baratang islands. This potentially indicates that population structure could have existed prior to the admixture detected, which we believe is a result of very recent processes fuelled by inter-island trade of food, coconut and banana saplings. Therefore, we considered the ‘North’ and ‘South’ clusters (Fig. 2) to test population divergence and admixture events at different points of time in the history of the Andaman day gecko populations.

Modelling the evolutionary history of the two clusters of the Andaman day gecko using ABC excluded a recent admixture between the North and South clusters resulting in a panmictic population across the sampled Andaman Islands (K = 1). The two supported models agree with respect to the time period in which the current pattern of weakly defined North and South clusters was formed. Scenario I (Fig. 5) supports a divergence time in the previous interglacial period (~ 153,000 years ago), followed by admixture prior to the onset of the last glacial maximum (~ 27,600 years ago). Contrastingly, scenario IV supports a divergence estimate also prior to the onset of the last glacial maximum (~ 23,600 years ago). However, it was not possible to statistically differentiate between the admixture model or the vicariance model giving rise to the North and South clusters (see scenario I and IV, Fig. 5). One possible explanation for not being able to tease the two scenarios apart could be that the Andaman day gecko populations have a complex divergence and admixture history due to repeated rise and fall in sea levels. The current population genetic composition showing signs of weak North and South clusters would be a result of the last vicariant divergence/admixture event, and earlier patterns of genetic diversity or structure, in this hypothesis, could have been partly or completely overridden by this event. The last admixture/vicariant event detected in this study is estimated to have occurred prior to the onset of the rise in sea level^38^ in the Late Pleistocene. Considering the putative complexity of repeated vicariance and admixture events in the Pleistocene glacial cycles, even comprehensive genomic data may fail to reconstruct these demographic processes in detail.

Mitochondrial DNA revealed substantial divergence among haplotypes that is likely indicative of earlier diversification processes, and this is also supported by the rather early divergence event estimated in scenario I of the ABC models (Fig. 4). The presence of an exclusive haplotype on the Little Andaman with a distance of 5 bp from all other haplotypes provides a clear indication for a geographic component in these earlier processes (Fig. 5). A comparison with two other endemic gecko species revealed that the amount of mitochondrial variation across the Andaman Islands is similar for the Andaman day gecko and the Andaman giant gecko, but phylogeographic structure is more obvious in the latter. The Andaman bent-toed gecko has a much larger mitochondrial variation, but an equally strong phylogeographic structure as observed by the distribution of haplotypes detected and number of mutational steps between haplotypes (Fig. 5). Considering these results, we hypothesize that the phylogeographic patterns in populations of the Andaman day gecko also arose by vicariance, but recent events have subsequently blurred this original phylogeographic signal. This may include repeated vicariance/admixture during glacial cycles as discussed, but also anthropogenic influences.

In comparison with the Andaman day gecko, the other two species included in this study show haplotype networks that are more typical of geographically structured populations with limited gene flow and longer isolation (Fig. 5), even in the Greater Andaman Islands. A study on the Andaman Keelback (*Fowlea tytleri*) also detected structured populations through mitochondrial phylogeography^13^. However, all four species, including the Andaman Keelback, show unique haplotypes on Little Andaman, reflecting more restricted gene flow between Little Andaman and other islands (Fig. 5). A study on bats of the Andaman Islands also found unique mitochondrial haplotypes on Little Andaman Island in three species^12^. However, considering the microsatellite allelic diversity and distribution in island populations of the Andaman day gecko, we speculate that haplotypes from other islands could be present in Little Andaman and could be detected through more extensive sampling. But the mitochondrial haplotype network does not reflect complete admixture similar to the microsatellite markers, it rather reflects ongoing admixture as several private haplotypes still persist in island populations (Fig. 5). Further, the lack of median joining vectors in the COI network also confirms extensive sampling and that all ancestral haplotypes linking island populations have been detected.

Although mitochondrial allelic diversity is high in the populations of the Andaman day gecko, their geographic distribution is poorly structured compared to other reptile species included in this study (Fig. 5). We hypothesize that the differences in population structure of the three species of geckos are caused by a combination of differences in their ecological traits and susceptibility to human-mediated dispersal. The Andaman day gecko is active by day and is primarily found in high densities in banana, coconut, and areca nut plantations. On the other hand, the Andaman bent-toed gecko is vespertine and is mostly ground dwelling, only climbing tree trunks up to 1–2 m. The Andaman giant gecko can be found inhabiting the canopy of tall, evergreen trees and inside human constructed structures on high walls or tiled ceilings. Due to the direct association of the Andaman day gecko with plants, reproductive parts of plants (banana inflorescences and fruit clusters) and trees in plantations, individuals of this species and their eggs could be dispersed more easily through sapling or food trade between islands.

Differences in ecological traits between the Andaman day gecko and the other species support the hypothesis that early human settlers of the Andamans could have facilitated dispersal of the Andaman day gecko, especially within the Greater Andaman Islands. The Jarawa tribe for example, are known for their extensive use of plants including forest palms^39^ which could be used by the Andaman day gecko. The Little Andaman tribe, the Onge people however were restricted to the Little Andaman and surrounding islands^36^. Therefore, the larger expanse of ocean, higher geographical distance, and lack of movement in early human settlers could have all influenced the mitochondrial distinctness of the Little Andaman Island populations.

Notably, our study shows the possible role of modern human colonizers in the observed genetic diversity and distribution of the Andaman day gecko due to the introduction of plantations and increased movement between islands. This is supported both by the expanding population demographics estimated by ABC models (Table 3) and a completely admixed population detected as the most probable cluster using microsatellite markers (K = 1) (see Supplementary S2, Figs. 4, 5 online). A recent study also reported the Andaman day gecko in the Nicobar Islands for the very first time^40^. Production of food for human consumption, such as banana and cash crops like betel nut and coconut have certainly provided an easy access to alternative high energy food sources that facilitated the existence of high densities of Andaman day gecko in plantations (see^21^). Such alternative food sources for animals, which are a result of human induced changes can be considered as anthropogenic food subsidies, and may have altered food chains and ecosystems^41^. Individuals of the Andaman day gecko have been spotted among saplings in boats and ships^21^ and some individuals/eggs could get transported along with  banana fruits to the local markets. Such high densities of *Phelsuma* geckos are not seen in only the Andaman Islands; a study in Madagascar on other species of *Phelsuma* has shown that the encounter rate for three species was higher in fragmented and agricultural lands than in forests^22^. This study concluded that adaptive generalist day gecko species may benefit from anthropogenic disturbances while specialized species experience negative impacts of habitat fragmentation. The Andaman day gecko qualifies as a generalist species adapting to different habitats and microhabitats despite anthropogenic stress. The impacts of anthropogenic food subsidies on the Andaman day gecko remains to be explored; however, our ABC models do suggest that the population sizes have expanded (Table 3).

The Andaman Islands present a system of continental islands that are proximate to mainland Southeast Asia, yet harbour high levels of endemism^5^. In this study, we show that the only Malagasy component of the Andaman Islands is one of the oldest species of the genus *Phelsuma* (Fig. 2). The Andaman day gecko is characterized by high levels of allelic diversity both at mitochondrial and nuclear microsatellite markers, but a distinctly lower phylogeographic structure than that of other co-occurring geckos. Our results agree with Heaney’s^1^ assumption that the amount of gene flow and dispersal varies between superficially similar, co-distributed taxa and that the differences in their ecology can create substantial differences in gene flow between neighbouring islands. ABC models favour change in sea levels having strongly influenced the admixture or vicariance events in the past. But a single expanding panmictic population of the Andaman day gecko detected in this study could be a result of more recent anthropogenic changes to their habitats and human mediated dispersal across different islands of the Andaman Archipelago. These factors have not only resulted in demographic expansion but have also potentially blurred signals of a putative geographical differentiation that may have arisen through earlier processes.

## Methods

### Study area and sampling

The Andaman Islands are located between 13.66^o^N, 93.00^o^E and 10.54^o^N, 92.46^o^E and receive an annual rainfall of 3,000–3,500 mm^42^. The topography is flat with low elevation hills, except the Mount Harriet National Park (383 m) in South Andaman Island and the Saddle Peak National Park (732 m) on North Andaman Island.

**Figure 6:**
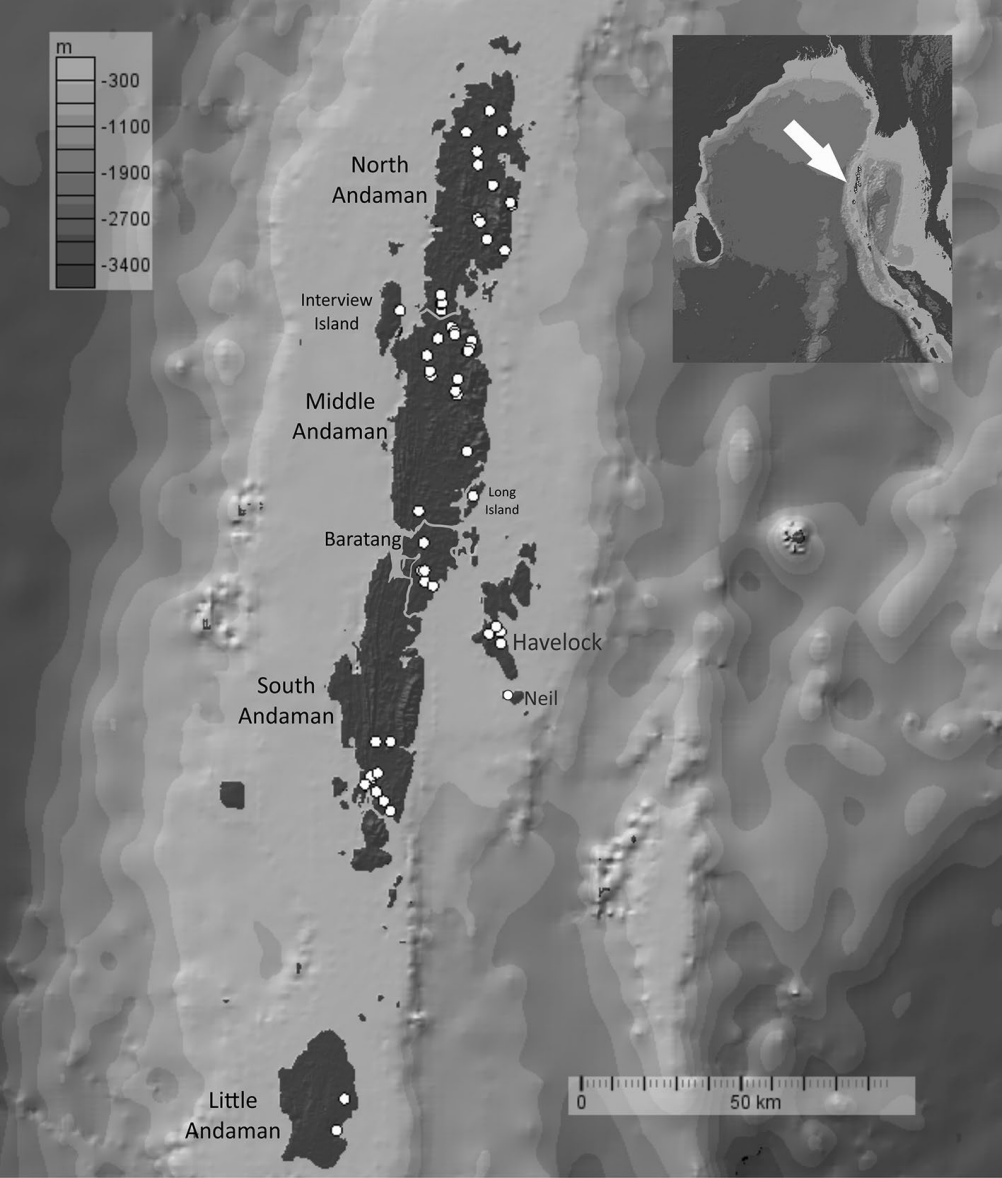
Sampling map of the Andaman day gecko (*Phelsuma andamanensis*) on the Andaman Islands. White points are the localities sampled for this study. Map produced in GeoMapApp (https://www.geomapapp.org/) and labelled using CorelDraw Home & Student, X7 (https://www.coreldraw.com).

We sampled the Andaman day gecko (*Phelsuma andamanensis*), Andaman bent-toed gecko (*Cyrtodactylus rubidus*) and the Andaman giant gecko (*Gekko verreauxi*) from 10 islands of this archipelago, including two non- human inhabited islands (Fig. 6). Study permits to work on these islands and to sample geckos were issued by the Department of Environment and Forests, Andaman and Nicobar Islands (Permit No.: CWLW/WL/134(A)/517), and this permit included all aspects of field work and sample collection. Field sampling was conducted between October 2016 and May 2017 which spans the North-east monsoon period (October–December) and the summer season (Feb–April) in the Andaman Islands. Sampling for the Andaman day gecko was primarily carried out in betel nut, coconut and banana plantations as the forests generally encompassed tall evergreen species and this species occurs at much lower densities in forests^21^. The sampling sites were distributed throughout the major islands excluding the Jarawa tribal reserve. The reserve, home to the Jarawa tribe, spans just over 1,000 sq. km including areas in South and Middle Andaman Islands^43^ and access is prohibited. The elevation in sampled points ranged from ca. 1 to 75 m above sea level. A team of two to four personnel conducted opportunistic visual encounter surveys between 8:00 and 11:00 h and 14:00 to 16:00 h. Upon locating and capturing the Andaman day gecko, we recorded the geographical location (using a Garmin GPSMAP 64) with an accuracy of 2–5 m. We used a bamboo stick or other wooden equipment to tap the lizards as a result of which they would drop (autotomize) parts of their tails. We collected their tails and stored them in 99.99% laboratory grade ethanol. We noted the sex of individuals without capture as this species is sexually dichromatic with males showing a turquoise blue tail (Fig. 1). Therefore, we avoided capturing individuals ensuring minimal stress to the animal. In the few cases where individual capture was necessary, we used sterilized surgical steel scissors to cut the tail tip (up to 15 mm from the tip). After collecting the tissue, we sterilized the open wound and allowed the individual to settle for a few minutes before releasing them at the capture site. The time taken in handling individuals did not exceed 3–5 min. Note that as in most lizards, the autotomized/cut tail tips will naturally regenerate. The tissue samples were deposited at the Centre for Ecological Sciences, Indian Institute of Science, Bangalore. This handling protocol was approved by the Department of Environment and Forest, Andaman and Nicobar Islands Research Advisory Committee and adheres to international Animal Care and Use guidelines. All the collected samples, with field labels and GPS location of collection are tabulated. (see Supplementary S1, sheet 1 online).

### Phylogenetic position of the Andaman day gecko

Previously published molecular marker datasets were downloaded from NCBI (see Supplementary S1, sheet 2 online) and new markers were added by amplification through PCR and sequencing carried out specifically for this study. As our aim was to show the relative position of the Andaman day gecko in the genus, we only selected one representative species from each of the previously recognized *Phelsuma* clades^14^. In addition to the published data, we amplified portions of the genes for KIAA1239 (a long stretch of DNA coding for proteins involved in several cellular processes) with the primers KIAA1239-F1, KIAA1239-R1, KIAA1239-NF1 and KIAA1239-NR1; Spastic Ataxia Of Charlevoix-Saguenay or Sacsin (SACS) using primers SACS-F1, SACS-R1, SACS-NF1 and SACS-NR1, and Titin (TTN) with the primers TTN-F1, TTN-R1, TTN-NF1 and TTN-NR1^44^. All the three additional markers were amplified using a two-step, nested PCR^44^. Additional sequences of portions of the genes for Phosducin (PDC), Brain Derived Neurotrophic Factor (BDNF), Acetylcholinergic receptor M4 (ACM4) and Cytochrome c oxidase subunit I (COI) were amplified for species to fill gaps in the dataset available online (see Supplementary S1, sheet 2 online). We utilized the primers PHOF2 and PHOR1^45^ to amplify a portion of the PDC gene, BDNF-F2 and BDNF-R2^46^ for BDNF, tg-F and tg-R primers^47^ for ACM4 and the afore mentioned primers for COI. A dataset containing 15 taxa, including outgroups and a concatenated sequence totalling 9,278 bp was used to reconstruct the phylogenetic relationships presented in this study. The phylogenetic relationships among the selected taxa were reconstructed using a GTR model of sequence evolution, with the thorough bootstrap algorithm of 10 runs and 10,000 iterations in RaxML^48^.

### Population genetics of the Andaman day gecko based on microsatellite markers

We sequenced 15 microsatellite markers from 141 individuals of this species sampled across 10 islands of the Andaman Archipelago. A tail clip sample of one individual of the Andaman day gecko was used for the development of species-specific microsatellite markers. This process was carried out in the Sequencing Genotyping Facility at the Cornell Life Sciences Core Laboratory Centre (CLC), U.S.A. (commercial facility). To summarize the protocol, genomic DNA was extracted from the tail tip tissue and restriction enzymes were used to digest the DNA and Illumina TruSeq adapters were ligated to the digested DNA. Through magnetic capture and hybridization of biotinylated repeat probes (representing two unique dimers, five unique trimers, seven unique tetramers and two unique pentamers), the fragments were enriched for microsatellites. The DNA fragments were then amplified and barcoded by Polymerase Chain Reaction (PCR), then sequenced in the Illumina MiSeq sequencer (2 X 250 bp paired reads). The library consisted of minimum consecutive perfect repeat lengths of at least six (12 bp) for dimers and at least five for trimers, tetramers, pentamers and PCR products whose size was between 150–500 bp. The full library generated using the above method is given (see Supplementary S3 online), but the microsatellite markers used in this study were further shortlisted based on the following categories: (1) tetrameric, (2) repeat motif between 10 and 15, (3) less than 1,000 reads, as deep coverage could indicate multiple copies and (4) GC content of 50 (Table 4)^49^. The microsatellite marker amplification was done using the nested protocol with the following steps: 900 s of initial denaturation at 94 °C, 30 cycles of 94 °C (30 s), 60 °C (45 s), 72 °C (45 s), followed by 8 cycles of 94 °C (30 s), 53 °C (45 s), 72 °C (45 s), and a final elongation step of 600 s at 72 °C^50^. 25 markers were shortlisted after the filtering steps (see Supplementary S3 online) and among the 25 markers, 15 markers resulted in reliable bands on the Agarose gel electrophoresis step. These 15 markers were further used to amplify microsatellite alleles from all samples of the Andaman day gecko available for this study.

Total genomic DNA was extracted from the tissue samples using proteinase K digestion (10 mg/ml concentration) followed by a standard salt-extraction protocol^51^. We amplified all microsatellite markers following the nested protocol of Schuelke (2000), but instead of using the M13 sequence we linked the Illumina sequencing primer sequence FAM (ACACTCTTTCCCTACACGACGCTCTTCCGATCT) to all forward primers. This is a modified M13 approach. We diluted the PCR product with 15 μl of RNase-free water and pipetted 1 μl diluted product to a genotyping plate. We then added 15 μl of Genescan 500–ROX size standard (Applied Biosystems) and analysed fragments in ABI 3130xl Genetic Analyzer. Microsatellite data were scored by creating customized data panels in the program Gene marker v1.95 (https://softgenetics.com/GeneMarker.php). For a subset of samples ~ 50, we repeated the PCR 3 times and they were carried out by 3 different persons to check and compare the peaks to ensure repetition gave consistent peaks. Data panels were created by observing concordant peaks at different molecular weights across different samples for the same markers. Allele peaks were scored by the same person, with a time gap of minimum 7 days and the panels were made twice, independently and compared to observe differences. Scoring panels were created for each microsatellite marker by comparing resultant peaks across individual samples originating from different islands. After comparing the two panels and fixing one scoring panel for each marker, the alleles were scored twice independently. Microsatellite allele data obtained from 15 microsatellite markers were checked for levels of missing data and two markers were removed due to high proportion of missing data and inconsistency in peaks. 13 markers were used for further population genetic analyses (see Supplementary S2, sheet 1 online).

Sampled localities were plotted on a map using GeoMapApp^52^ and modified using CorelDraw student X (Corel Corporation ltd.) (Fig. 5). An Allele Sharing Matrix (ASM) was calculated for 13 microsatellite markers using the Microsatellite Toolkit package (developed by Stephen Park) implemented in Microsoft Excel (Microsoft Corporation INC.) (see Supplementary S4, sheet 2 online). With the microsatellite marker dataset, the software Cervus was used to test the presence of null alleles^53^. We tested if the 13 microsatellite markers used in this study were enough to identify true genetic diversity of the populations by conducting a power analysis as implemented in Poppr^54^. The 13 microsatellite markers used in this study were tested for Linkage disequilibrium implemented in the genepop package on the web^55^ using Fischer’s pairwise marker comparison across all sampled islands^56^. All microsatellite summary statistics were calculated in PopGenReport^57^and Adegenet^58^ implemented in R v3.5.2 (https://www.R-project.org/). For all markers, samples from six island populations were tested for Hardy–Weinberg equilibrium (HWE) using both Chi-squared and exact tests as implemented in Pegas package^59^. Population pairwise F_ST_, expected heterozygosity (He) and observed heterozygosity (Ho) were calculated in Microsatellite Analyzer (MSA)^60^. The F_IS_ statistic for each marker pair within each population was calculated in the R package, diveRsity^61^.

The program Structure 2.3.4^32^ was used to test the number of clusters of the Andaman day gecko using all 13 microsatellite markers that were amplified. The admixture model was used with correlated allele frequency settings and location priors were used to infer the number of clusters in the dataset. Structure was run for values of K from 1 to 6 with 2,000,000 steps of the MCMC algorithm and 50,000 steps as burn-in and repeating each estimate of K 10 times. Data from these runs were then summarized using Pophelper^62^ to calculate Evanno’s Delta K^63^. We removed the marker in which we detected null alleles, Pand9 and re-ran Structure to test if presence of null alleles has an impact on clustering patterns (see Supplementary S2 Fig. 3 online).

The best K identified using Evanno’s was K = 2, but we fear this was the case because Evanno’s K method cannot test for K = 1 as the best possible number of clusters. Evanno’s method is also known to identify K = 2 at the top level of hierarchical structure more frequently even when subpopulations are present^64^. Therefore, we also assess the K value which has the highest probability, Pr(K = k) implemented in Clumpak^65^ (see Supplementary S2 Fig. 4 in online). In addition, we tested population structure using the program rmaverick^33^, as it is known to overcome some of the limitations that are posed by Structure^64^. We tested both admixture and non-admixture models (see Supplementary S2 Fig. 4 online) and with K varying from 1 to 6 (representing large sampled islands) to test for the best value of K (see Supplementary S2 Fig. 4 online). Further analyses downstream were based on the second-best partition solution for the data (K = 2).Structure runs were set to K = 2 with admixture model and location prior with correlated allele frequency model was used to run 20 iterations, each with 50,000 MCMC burn in period and 2,00,000 MCMC chains. Results were summarized using Clumpp^66^ implemented in the software Pophelper.

ASM from microsatellite markers and pairwise uncorrected p distance from mitochondrial COI sequences were plotted on a map using Mapi^67^, an exploratory tool to map distance matrices. Mapi is also used to identify areas of highest genetic differentiation, which is useful in interpreting the role of geographical barriers in the distribution of genetic diversity.

### Testing best fit population demographic models through Approximate Bayesian Computation (ABC)

Considering the two weak clusters detected using microsatellite markers, we tested for different population demographic scenarios using Approximate Bayesian computation (ABC) using DIY-ABC v2.0^68^. The models tested for four alternative demographic models to determine which model explains the partition of individuals into North and South clusters. All the four models consisted of an ancestral split into two populations, with the first three models also including an admixture event between both clusters (i.e. bidirectional) and with equal contribution of each cluster but different with respect to the time when the admixture occurred. The fourth scenario was a simpler scenario where an ancestral population splits into two to gives rise to the North and South clusters (Fig. 5). Mutation rates for the mitochondrial DNA sequence data and the microsatellite data were left as default with the mitochondrial DNA sequence mean mutation rate per site varying between 10^–9^ and 10^–4^ following the Kimura 2 Parameter model (1980). The microsatellites’ mutation rate was set to a generalized stepwise mutation model^69^ with a gamma prior distribution for each locus mutation rate varying between 10^–7^ and 10^–2^, and with a mean across loci mutation rate defined with a uniform prior between 10^–6^ and 10^–2^. Uniform prior distributions were also used for the estimation of effective population sizes and for the time of divergence or the time of admixture. Effective population sizes could vary between 10 and 4,000,000. The time of divergence between the populations could vary between 35,000 and 4,00,000 years ago for scenarios I–III, and between 10 years and 60,000 years ago for scenario IV. The time of admixture for scenario I was allowed to vary between 2,000 to35,000 years ago, while for scenario II, it was allowed to vary between 501 to 6,000 years ago and for scenario 3, it was allowed to vary between 10 to 500 years ago. The lower limit of 35,000 years was decided based on the sea level in the Late Pleistocene^38^. At ~ 35,000 years ago, the sea level was 65 m below the current sea level^38^, after which the sea level reduced to ~ 135 m lower than present, until up to ~ 20,000 years ago. The upper limit of 4,00,000 was chosen as a conservative maximum estimate because of the inclusion of mitochondrial markers that are slowly evolving compared to the microsatellites. We allowed the Scenario I to have an extremely broad range for the admixture time (35KYA–2KYA) where clusters North and South could have been the result of varying sea levels. For the Scenario II, admixture time range was decided based on the current glacial maxima (rise in sea level ended ~ 6,000 years ago)^38^ and the lower age limit was to ensure no role of modern humans. In Scenario III, we exclusively considered the role of modern human settlers in the formation of North and South clusters (500–10 YA). Admixture rate was fixed at 50:50 contribution of each of the ancestral populations. The summary statistics used to compare simulations and observed data from the mitochondrial DNA were: (1) estimated for each cluster: the number of haplotypes, number of segregating sites, mean number of pairwise differences, variance in the number of pairwise differences, the number of private segregating sites, the mean number of the rarest nucleotide segregating and the variance of the number of rarest nucleotide segregating; (2) estimated across clusters: number of haplotypes, number of segregating sites, the average of the mean number of pairwise differences within each cluster as well as the mean number of pairwise differences between the two clusters, and the Fst. The summary statistics used to compare simulations and observed data from the microsatellites markers were: (3) the mean number of alleles, mean genetic diversity (both measured for each population and across the two populations), and F_ST_. We used DIY-ABC’s Principal Component Analysis (PCA) to pre-evaluate if the parameters of prior distributions set for the simulations could recover the observed data using 10,000 random points equally distributed across the four scenarios. Among the four scenarios tested, the best fit scenario was identified using polychotomic weighted logistic regressions on the 1% closest simulations to the observed data among the 400,000 simulations carried out, with a dependent and an independent variable. Proportion of support for the scenario was the dependent variable and the difference between observed and simulated data summary statistics was the independent variable used.

### Comparative phylogeography of Andaman endemic Gekkonidae

We sequenced mitochondrial COI sequences from 123 individuals of the Andaman day gecko sampled across 10 islands of the Andaman Archipelago to obtain a detailed mitochondrial phylogeography of this species. A section of the gene for mitochondrially encoded Cytochrome C Oxidase Subunit I (COI) was amplified using the primers LCO1490 (forward) and HCO2198 (reverse)^70^ at an annealing temperature of 49 °C. For comparative mitochondrial phylogeography, a segment of the 16S rRNA gene was amplified from representative samples of the Andaman day gecko (*P. andamanensis*), Andaman bent-toed gecko (*Cyrtodactylus rubidus*) and the Andaman giant gecko (*Gekko verreauxi*). We generated 463 bp 16S sequences from 17 individuals of the Andaman day gecko, 480 bp sequences from 13 samples of the Andaman bent-toed gecko and 516 bp sequences from 12 individuals of the Andaman giant gecko. 16S rRNA samples were amplified using the primers 16Sar-L and 16Sbr-H^71^. All obtained PCR products were purified by Exonuclease I and Shrimp Alkaline Phosphatase digestion and sequenced on a 3130xl genetic analyser (Applied Biosystems) using Big Dye v3.1 cycle sequencing chemistry. Chromatograms were quality checked by eye, trimmed for low-quality stretches and errors corrected using Codon Code Aligner (v5.1.5, Codon Code Corporation). All newly determined sequences have been deposited in GenBank (accession numbers MT677531-MT679993; includes new sequences used for phylogenetic analysis of the genus *Phelsuma*, see above; details in see Supplementary S1, sheet 3 online). All pair-wise sequence divergence distances presented in the manuscript are uncorrected *p* distances generated in MEGA 7.0^72^ (see Supplementary S4, sheet 1 online).

The final alignment of COI gene consisted of 403 bp after trimming the ends for haplotype analyses. We calculated the number of haplotypes/alleles, number of variable sites in the alignment, nucleotide diversity (π), the average number of nucleotide differences (k) and haplotype diversity (H_d_) in DnaSP 10.01^73^. The same steps were followed to reconstruct haplotype networks with the 16S sequences of all three species.

We tested if the number of haplotypes detected with 17 samples of the Andaman day gecko for the marker 16S (6 haplotypes) was significantly different from the number of haplotypes detected for the COI marker. For this purpose, we estimated the probability of identifying 6 haplotypes among 10,000 random samples with replacement of 17 COI sequences sampled among the 123 sequences described here (see Supplementary S2, Fig. 2 online). We generated a Roehl Data Format (RDF) file in DnaSP and calculated Median Joining networks^74^ in the software NETWORK 5.0 (fluxus-engineering.com). Networks were colour-coded geographically using Corel Draw Student X (Corel Corporation). Pairwise Nei’s GST distances between islands populations were calculated with the packages mmod^75^ (Table 2).

## Supplementary information

Supplementary file1 (XLSX 39 kb)

Supplementary file2 (DOCX 874 kb)

Supplementary file3 (XLSX 17640 kb)

Supplementary file4 (XLSX 181 kb)
