# Supplementary material for: The Andaman day gecko paradox: an ancient endemic without pronounced phylogeographic structure
Source: Sci Rep. 2020 Jul 16;10:11745. doi: 10.1038/s41598-020-68402-7 (PMC7367275; doi:10.1038/s41598-020-68402-7)
Supplement: Supplementary file 2 — Supplementary file2 (DOCX 874 kb) [file 41598_2020_68402_MOESM2_ESM.docx]

**The Andaman day gecko paradox: an ancient endemic without pronounced phylogeographic structure**

Ashwini V. Mohan^1,2 *^, email id: ashwinivm30@gmail.com

Pablo Orozco-terWengel^3^, email id: orozco-terwengelpa@cardiff.ac.uk

Kartik Shanker^2^, email id: kshanker@gmail.com

Miguel Vences^1^, email id: m.vences@tu-braunschweig.de

| Species | Genetic marker | No. of samples | No. of alleles | No. of Median vectors | No. of Private alleles | No. of shared alleles | Maximum uncorrected *p* distance among alleles |
| --- | --- | --- | --- | --- | --- | --- | --- |
| *Phelsuma andamanensis* | COI | 123 | 25 | 0 | 17 | 8 | 1.6% |
| *Phelsuma andamanensis* | 16S | 17 | 6 | 1 | 4 | 2 | 0.9% |
| *Cyrtodactylus rubidus* | 16S | 13 | 10 | 5 | 10 | 0 | 1.83% |
| *Gekko verreauxi* | 16S | 12 | 4 | 1 | 3 | 1 | 1.16% |
| *Xenochrophis tytleri* | 16S | 17 | 7 | 0 | 6 | 1 | 0.71% |

Table 1: Summarizing mitochondrial haplotype information from all the four species compared in this study.

| Locus | Locus | NA | MA | BA | HA | SA | LA |
| --- | --- | --- | --- | --- | --- | --- | --- |
| Pand2 | Pand6 | 1.00 | 1.00 | _ | _ | _ | _ |
| Pand2 | Pand8 | 0.60 | 1.00 | _ | _ | _ | _ |
| Pand6 | Pand8 | 1.00 | 1.00 | _ | _ | 0.02 | _ |
| Pand2 | Pand10 | 1.00 | 1.00 | _ | _ | _ | _ |
| Pand6 | Pand10 | 1.00 | 1.00 | 1.00 | 0.17 | 1.00 | _ |
| Pand8 | Pand10 | 1.00 | 1.00 | _ | _ | _ | _ |
| Pand2 | Pand12 | 1.00 | 1.00 | _ | _ | _ | _ |
| Pand6 | Pand12 | 1.00 | 1.00 | 1.00 | 0.10 | 1.00 | _ |
| Pand8 | Pand12 | 1.00 | 1.00 | _ | _ | 1.00 | _ |
| Pand10 | Pand12 | 1.00 | 1.00 | 1.00 | 0.18 | 1.00 | _ |
| Pand2 | Pand13 | 1.00 | 1.00 | _ | _ | _ | _ |
| Pand6 | Pand13 | 1.00 | 0.30 | _ | 0.11 | 1.00 | _ |
| Pand8 | Pand13 | 1.00 | 1.00 | _ | _ | 1.00 | _ |
| Pand10 | Pand13 | 1.00 | 1.00 | _ | 0.06 | 1.00 | _ |
| Pand12 | Pand13 | 0.29 | 1.00 | _ | 0.11 | 0.03 | _ |
| Pand2 | Pand15 | 1.00 | 1.00 | _ | _ | _ | _ |
| Pand6 | Pand15 | 1.00 | 1.00 | _ | 0.12 | 1.00 | _ |
| Pand8 | Pand15 | 1.00 | 1.00 | _ | _ | 1.00 | _ |
| Pand10 | Pand15 | 1.00 | 1.00 | _ | 0.11 | 1.00 | _ |
| Pand12 | Pand15 | 0.18 | 1.00 | _ | 0.23 | 1.00 | _ |
| Pand13 | Pand15 | 1.00 | 1.00 | _ | 0.08 | 1.00 | _ |
| Pand2 | Pand16 | 0.26 | 0.14 | _ | _ | _ | _ |
| Pand6 | Pand16 | 1.00 | 0.69 | 1.00 | 0.27 | 1.00 | _ |
| Pand8 | Pand16 | 0.14 | 0.50 | _ | _ | 1.00 | _ |
| Pand10 | Pand16 | 1.00 | 1.00 | 1.00 | 0.36 | 1.00 | _ |
| Pand12 | Pand16 | 0.49 | 1.00 | 1.00 | 0.53 | 1.00 | _ |
| Pand13 | Pand16 | 0.16 | 0.05 | _ | 0.19 | 1.00 | 0.29 |
| Pand15 | Pand16 | 1.00 | 1.00 | _ | 0.40 | 1.00 | _ |
| Pand2 | Pand17 | _ | 1.00 | _ | _ | _ | _ |
| Pand6 | Pand17 | 1.00 | 1.00 | _ | 0.09 | _ | _ |
| Pand8 | Pand17 | 1.00 | 1.00 | _ | _ | _ | _ |
| Pand10 | Pand17 | 1.00 | 0.21 | _ | 0.08 | _ | _ |
| Pand12 | Pand17 | 1.00 | 1.00 | _ | 0.09 | _ | _ |
| Pand13 | Pand17 | 1.00 | 1.00 | _ | 0.04 | _ | _ |
| Pand15 | Pand17 | 1.00 | 1.00 | _ | 0.09 | _ | _ |
| Pand16 | Pand17 | 1.00 | 1.00 | _ | 0.17 | _ | _ |
| Pand2 | Pand19 | 1.00 | 1.00 | _ | _ | _ | _ |
| Pand6 | Pand19 | 1.00 | 1.00 | _ | 0.06 | _ | _ |
| Pand8 | Pand19 | 1.00 | 1.00 | _ | _ | _ | _ |
| Pand10 | Pand19 | 1.00 | 1.00 | _ | 0.08 | _ | _ |
| Pand12 | Pand19 | 1.00 | 1.00 | _ | 0.11 | _ | _ |
| Pand13 | Pand19 | 1.00 | 0.65 | _ | 0.04 | _ | _ |
| Pand15 | Pand19 | 1.00 | 1.00 | _ | 0.09 | _ | _ |
| Pand16 | Pand19 | 0.06 | 0.16 | _ | 0.19 | _ | _ |
| Pand17 | Pand19 | 1.00 | 1.00 | _ | 0.04 | _ | _ |
| Pand2 | Pand23 | 1.00 | 1.00 | _ | _ | _ | _ |
| Pand6 | Pand23 | 0.60 | 1.00 | 1.00 | 0.29 | _ | _ |
| Pand8 | Pand23 | 1.00 | 1.00 | _ | _ | _ | _ |
| Pand10 | Pand23 | 1.00 | 0.55 | _ | 0.23 | _ | _ |
| Pand12 | Pand23 | 1.00 | 1.00 | 1.00 | 0.33 | _ | _ |
| Pand13 | Pand23 | 1.00 | 1.00 | _ | 0.09 | _ | _ |
| Pand15 | Pand23 | 1.00 | 1.00 | _ | 0.27 | _ | _ |
| Pand16 | Pand23 | 0.43 | 1.00 | 1.00 | 0.62 | _ | _ |
| Pand17 | Pand23 | 1.00 | 1.00 | _ | 0.11 | _ | _ |
| Pand19 | Pand23 | 1.00 | 1.00 | _ | 0.13 | _ | _ |
| Pand2 | Pand4 | 1.00 | 1.00 | _ | _ | _ | _ |
| Pand6 | Pand4 | 1.00 | 1.00 | _ | _ | _ | _ |
| Pand8 | Pand4 | 1.00 | 1.00 | _ | _ | _ | _ |
| Pand10 | Pand4 | 1.00 | 1.00 | _ | _ | _ | _ |
| Pand12 | Pand4 | 1.00 | 1.00 | _ | _ | _ | _ |
| Pand13 | Pand4 | 1.00 | 1.00 | _ | _ | _ | _ |
| Pand15 | Pand4 | 1.00 | 1.00 | _ | _ | _ | _ |
| Pand16 | Pand4 | 1.00 | 1.00 | _ | _ | _ | _ |
| Pand17 | Pand4 | 1.00 | 1.00 | _ | _ | _ | _ |
| Pand19 | Pand4 | 1.00 | 1.00 | _ | _ | _ | _ |
| Pand23 | Pand4 | 0.00 | 1.00 | _ | _ | _ | _ |
| Pand2 | Pand9 | 1.00 | 1.00 | _ | _ | _ | _ |
| Pand6 | Pand9 | 1.00 | 1.00 | _ | _ | _ | _ |
| Pand8 | Pand9 | 1.00 | 1.00 | _ | _ | _ | _ |
| Pand10 | Pand9 | 1.00 | 1.00 | _ | _ | _ | _ |
| Pand12 | Pand9 | 1.00 | 1.00 | _ | _ | _ | _ |
| Pand13 | Pand9 | 1.00 | 1.00 | _ | _ | _ | _ |
| Pand15 | Pand9 | 1.00 | 1.00 | _ | _ | _ | _ |
| Pand16 | Pand9 | 1.00 | 1.00 | _ | _ | _ | _ |
| Pand17 | Pand9 | 1.00 | 0.16 | _ | _ | _ | _ |
| Pand19 | Pand9 | 1.00 | 0.15 | _ | _ | _ | _ |
| Pand23 | Pand9 | 1.00 | 0.22 | _ | _ | _ | _ |
| Pand4 | Pand9 | 1.00 | 1.00 | _ | _ | _ | _ |

Table 2: Pairwise microsatellite marker comparison to test presence of Linkage disequilibrium calculated using Genepop implementing Fisher’s method. Columns contain permutated p values for pairwise tests among North Andaman Island (NorA), Middle Andaman (MA), Baratang (BA), Havelock (HA), South Andaman (SA) and Little Andaman (LA) populations. No LD calculations were feasible for small island populations of Long, Interview and Neil islands. Significance of the test inferred using FDR corrected *p* value (*p* =_-_0.010121). A hyphen is shown for the pairwise comparisons where the linkage could not be estimated.

|  | chi^2 | df | Pr(chi^2>) | Pr,exact |
| --- | --- | --- | --- | --- |
| Pand2 | 384,2093 | 351 | 1,07E-01 | 0,6806 |
| Pand6 | 134,5354 | 120 | 1,72E-01 | 0,822 |
| Pand8 | 400,2127 | 300 | 9,41E-05 | 0,199 |
| Pand10 | 351,0031 | 325 | 1,54E-01 | 0,415 |
| Pand12 | 497,8007 | 406 | 1,22E-03 | 0,118 |
| Pand13 | 172,4645 | 136 | 1,88E-02 | **0,006** |
| Pand15 | 343,7425 | 325 | 2,27E-01 | 0,025 |
| Pand16 | 117,7154 | 91 | 3,13E-02 | 0,121 |
| Pand17 | 1286,031 | 1176 | 1,34E-02 | **0,01** |
| Pand19 | 105,684 | 136 | 9,75E-01 | 0,81 |
| Pand23 | 234,2445 | 190 | 1,59E-02 | 0,035 |
| Pand4 | 231,2758 | 253 | 8,33E-01 | 0,445 |
| Pand9 | 285,0727 | 171 | 9,99E-08 | **0,002** |

Table 3: Results from test for Hardy-Weinberg Equilibrium of all the 13 microsatellite markers used in this study; calculated using both χ2-test and exact test using the package pegas in R; highlighted p values after FDR correction with *p* = 0.015723

|  | MA | NorA | SA | HA | LA | BA |
| --- | --- | --- | --- | --- | --- | --- |
| Pand2 | 0,275 | 0,792 | 0,082 | 1 | 1 | 1 |
| Pand6 | 0,386 | 0,988 | 0,76 | 0,196 | 0,232 | 0,457 |
| Pand8 | 0,124 | 0,294 | 0,05 | 1 | 1 | 0,787 |
| Pand10 | 0,356 | 0,918 | 0,029 | 0,101 | 1 | 0,564 |
| Pand12 | 0,286 | **0,006** | 0,358 | 0,026 | 0,071 | 0,546 |
| Pand13 | 0,079 | 0,074 | 0,759 | 0,54 | 1 | 0,876 |
| Pand15 | 0,413 | 0,164 | 0,09 | 0,308 | 0,036 | 0,888 |
| Pand16 | 0,281 | 0,166 | 0,191 | 0,04 | 1 | 0,986 |
| Pand17 | **0,005** | 0,12 | 0,443 | 0,02 | 1 | 1 |
| Pand19 | 0,838 | 0,059 | 1 | 0,088 | 0,751 | 0,661 |
| Pand23 | 0,218 | 0,02 | 0,505 | 0,089 | 1 | 0,772 |
| Pand4 | 0,275 | 0,908 | 1 | 0,826 | 0,778 | 0,699 |
| pand9 | 0,041 | **0** | 1 | 1 | 0,589 | 1 |

Table 4: Results from test for Hardy-Weinberg equilibrium of microsatellite markers from all sampled islands populations of the Andaman Islands, results from PopGenReport package in R, highlighted p values after FDR correction with *p* = 0.009357

| Marker | Frequency of null allele |
| --- | --- |
| Pand2 | 0,01 |
| Pand6 | 0,00 |
| Pand8 | 0,02 |
| Pand10 | 0,02 |
| Pand12 | 0,05 |
| Pand13 | 0,03 |
| Pand15 | 0,03 |
| Pand16 | 0,04 |
| Pand17 | 0,03 |
| Pand19 | 0,01 |
| Pand23 | 0,03 |
| Pand4 | 0,04 |
| Pand9 | **0,16** |

Table 5: Table with estimated null allele frequencies estimated from Summers & Amos, 1997’s method applied in Cervus


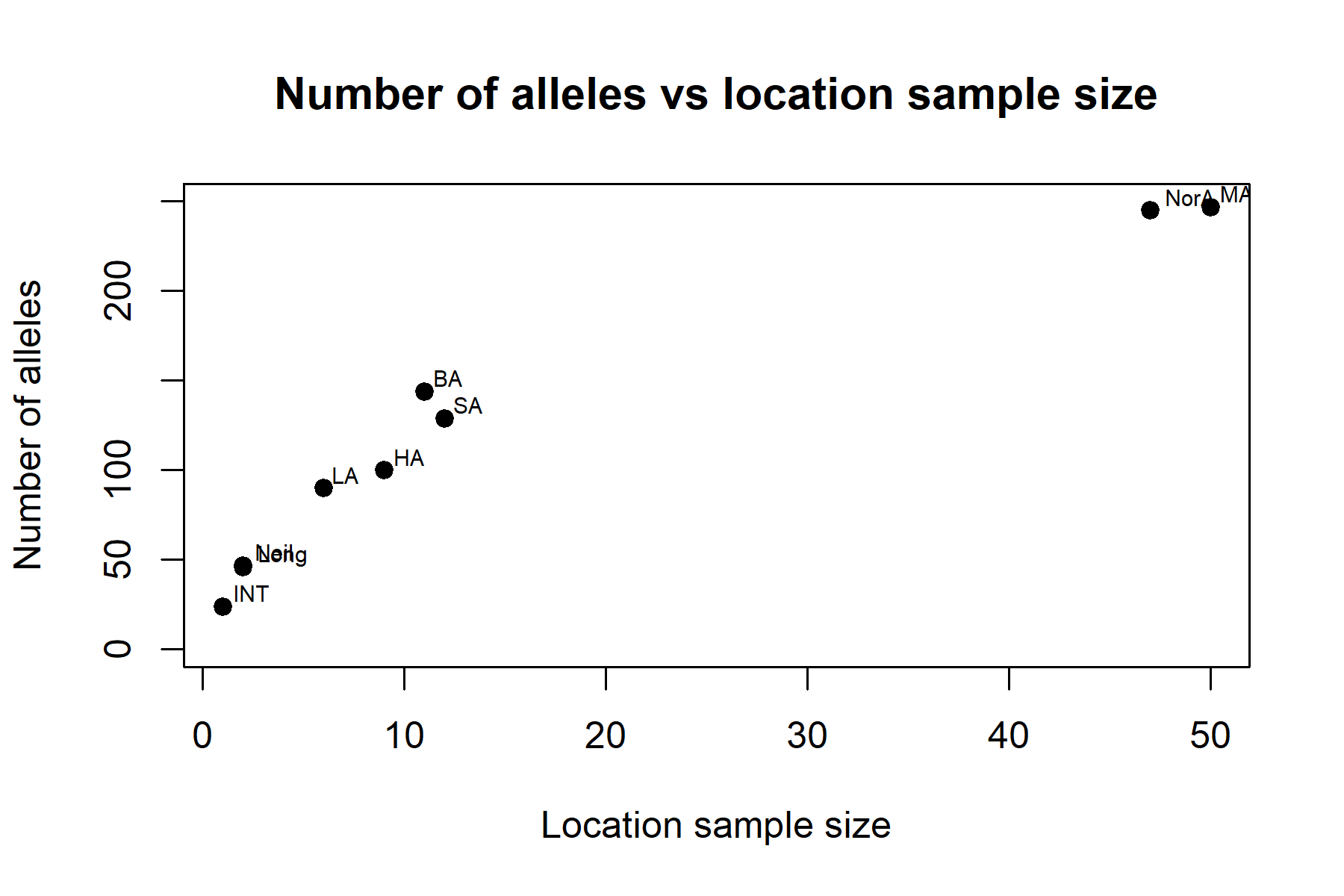


Figure 1: Plot depicting sample size from each island populations versus the number of alleles detected using microsatellite markers


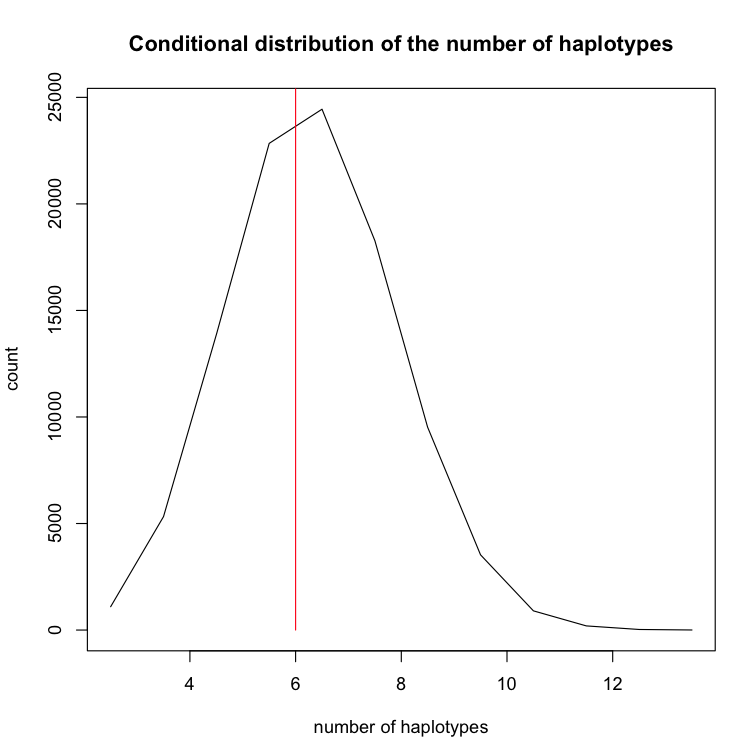


Figure 2: Histogram indicating where the observed 16S results from 17 samples of the Andaman day gecko (*Phelsuma andamanensis;* red line) falls within the distribution of random samples of 17 COI haplotypes randomly drawn from the set of 123 sequences in this paper’s dataset


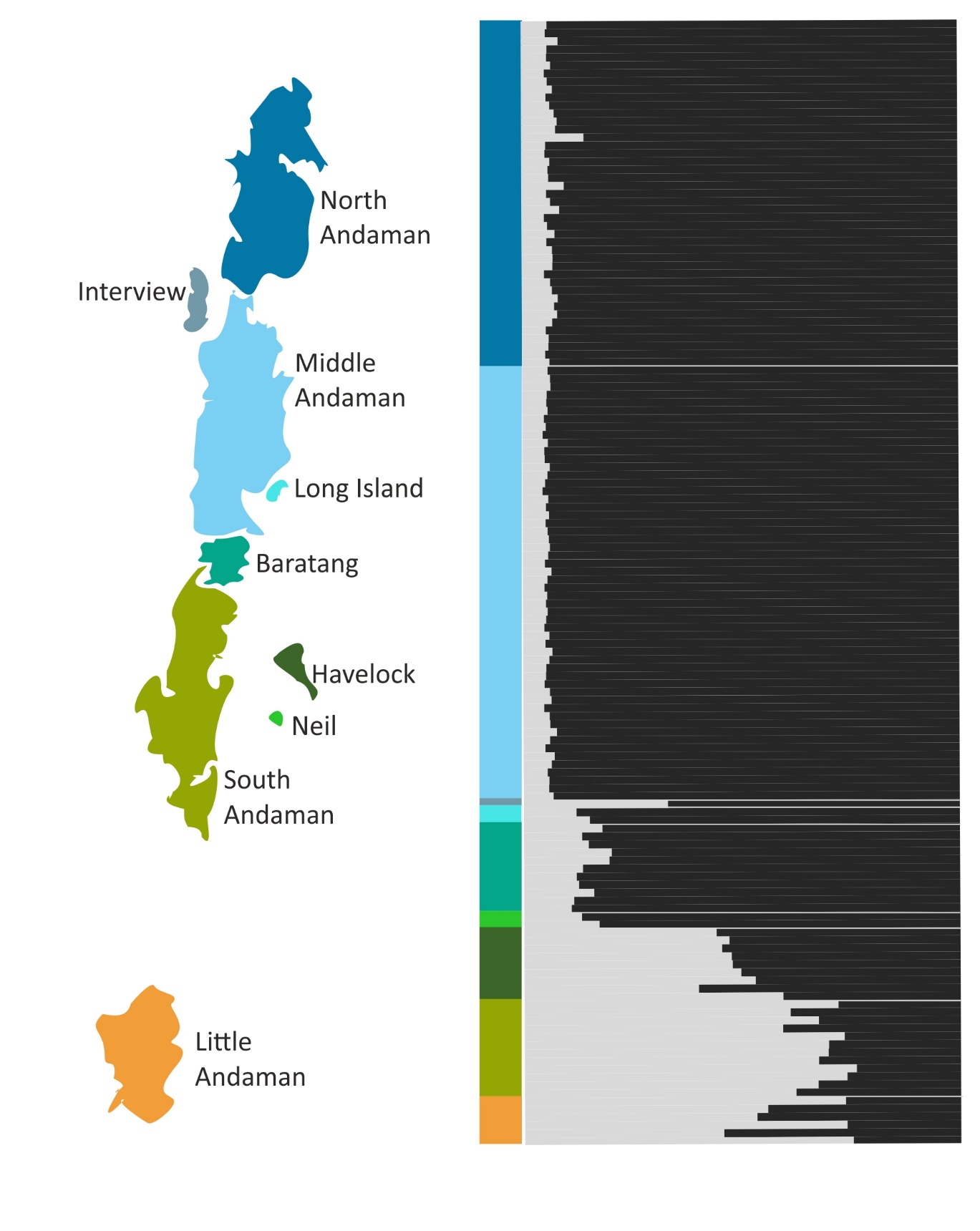


Figure 3: Bar plot generated using the results from the Bayesian clustering algorithm, STRUCTURE using 12 microsatellite markers, after removing marker in which null alleles were detected. White lines separate individuals sampled on different islands and island labels are colour coded in the corresponding blocks.


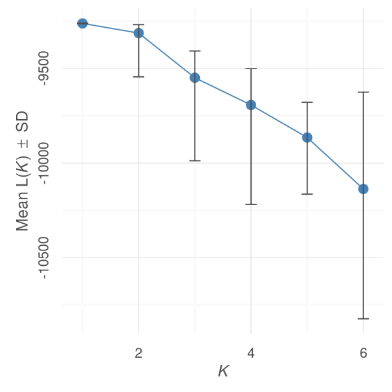

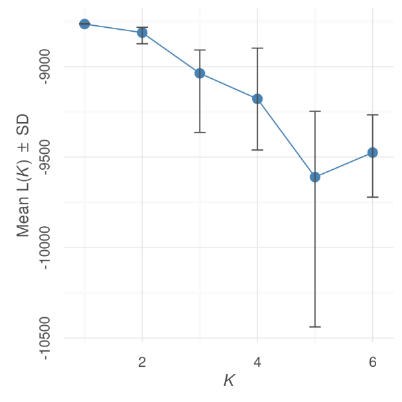

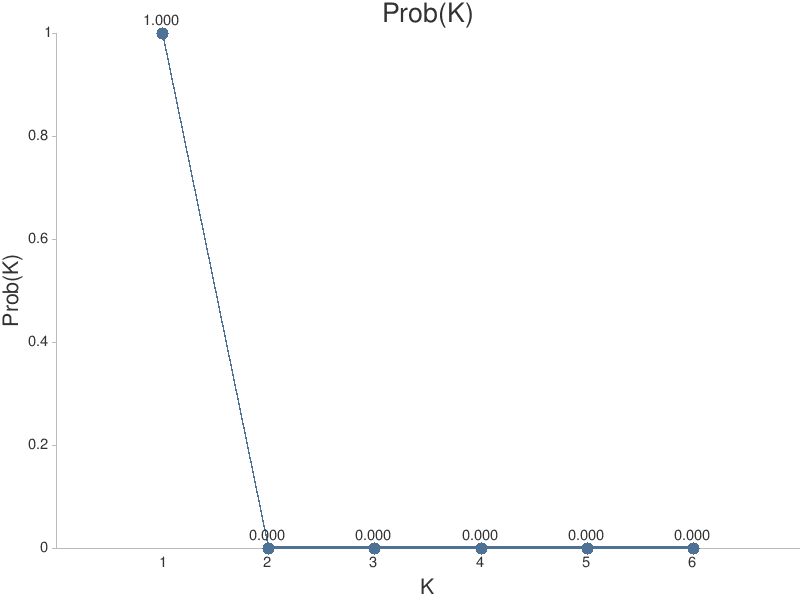


Figure 4: K value graphs from left: Delta K calculated from Structure run with all 13 microsatellite markers; Delta K calculated from Structure run with 12 microsatellite markers excluding a marker which showed presence of null alleles; the likelihood of K for each value of K (Ln Pr(X|K))


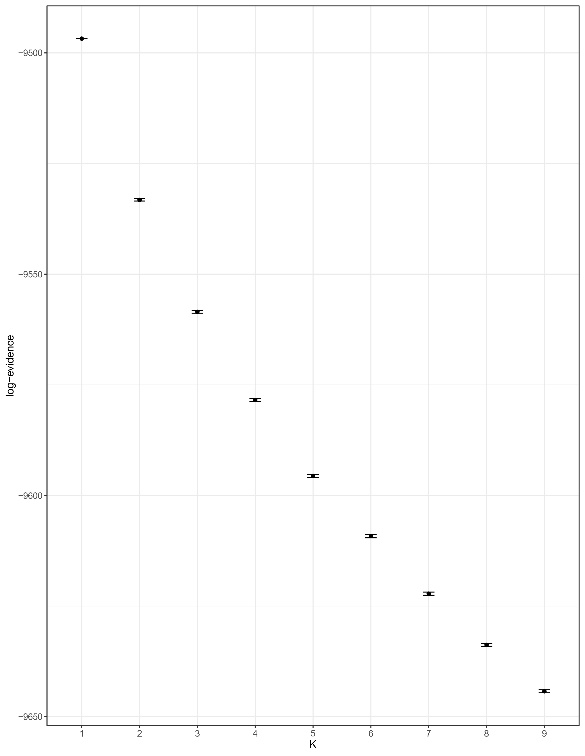

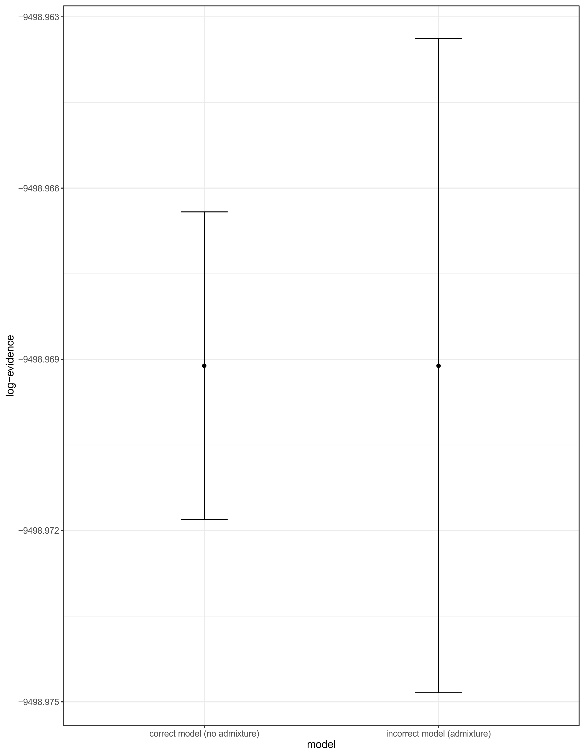


Figure 5: Best K value estimate obtained from rmaverick, run from a no-admixture model; Comparison between admixture and non-admixture model runs in rmaverick shows no clear support for one type.


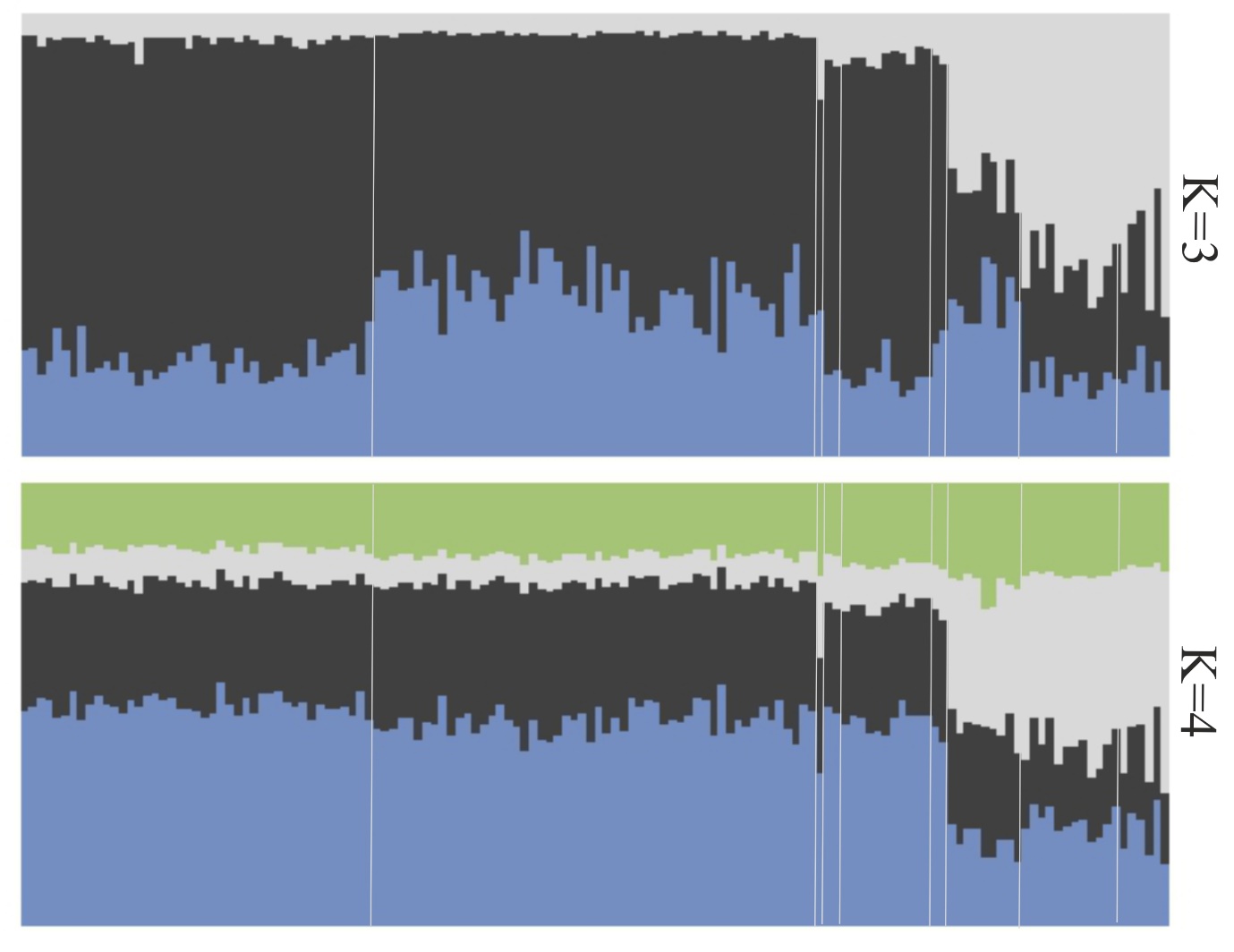


Figure 6: Bar plot generated using the results from the Bayesian clustering algorithm, STRUCTURE using 13 microsatellite markers, K was set to 3 and 4 respectively to show clustering patterns beyond K=2


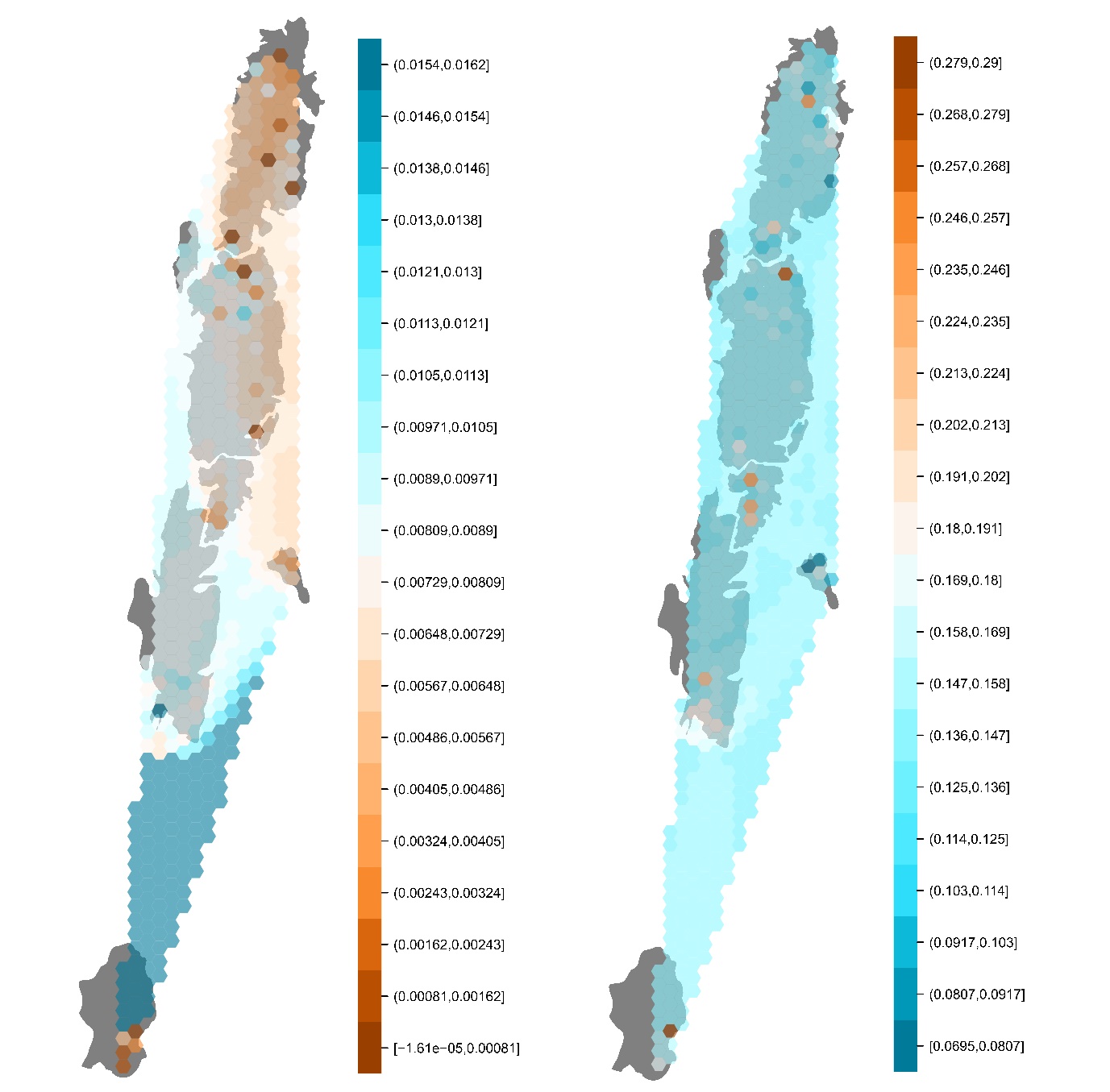


Figure 7: Genetic differentiation measures of microsatellite and mitochondrial data mapped using sampling localities of the Andaman day gecko (*Phelsuma andamanensis*). a. Microsatellite Allele Sharing Matrix mapped using sampling localities; brown sharing the highest number of alleles and blue the least; b. Genetic pairwise uncorrected *p* distances mapped on the Andaman Islands; brown being least uncorrected *p* distance and blue being the highest *p* distance.
